# Loss of murine Gfi1 causes neutropenia and induces osteoporosis depending on the pathogen load and systemic inflammation

**DOI:** 10.1371/journal.pone.0198510

**Published:** 2018-06-07

**Authors:** Sven Geissler, Martin Textor, Sabine Stumpp, Sebastian Seitz, Anja Lekaj, Sabrina Brunk, Sabine Klaassen, Thorsten Schinke, Christoph Klein, Stefan Mundlos, Uwe Kornak, Jirko Kühnisch

**Affiliations:** 1 Charité - Universitätsmedizin Berlin, corporate member of Freie Universität Berlin, Humboldt-Universität zu Berlin, and Berlin Institute of Health, Berlin-Brandenburg Center for Regenerative Therapies (BCRT), Berlin, Germany; 2 Charité - Universitätsmedizin Berlin, corporate member of Freie Universität Berlin, Humboldt-Universität zu Berlin, and Berlin Institute of Health, Julius Wolff Institute, Berlin, Germany; 3 Charité - Universitätsmedizin Berlin, corporate member of Freie Universität Berlin, Humboldt-Universität zu Berlin, and Berlin Institute of Health, Institute for Medical Genetics and Human Genetics, Berlin, Germany; 4 University Medical Center Hamburg-Eppendorf, Department of Orthopaedics, Hamburg, Germany; 5 Institut für Tierpathologie, Berlin, Germany; 6 Charité - Universitätsmedizin Berlin, corporate member of Freie Universität Berlin, Humboldt-Universität zu Berlin, and Berlin Institute of Health, Experimental and Clinical Research Center (ECRC), a joint cooperation between the Charité Medical Faculty and the Max-Delbrück-Center for Molecular Medicine, Berlin, Germany; 7 Charité - Universitätsmedizin Berlin, corporate member of Freie Universität Berlin, Humboldt-Universität zu Berlin, and Berlin Institute of Health, Department of Pediatric Cardiology, Berlin, Germany; 8 University Medical Center Hamburg-Eppendorf, Department of Osteology and Biomechanics, Hamburg, Germany; 9 Dr. von Hauner Children’s Hospital, Ludwig Maximilians University, Department of Pediatrics, Munich, Germany; 10 Max Planck Institute for Molecular Genetics, FG Development & Disease, Berlin, Germany; Universite de Nantes, FRANCE

## Abstract

Gfi1 is a key molecule in hematopoietic lineage development and mutations in *GFI1* cause severe congenital neutropenia (SCN). Neutropenia is associated with low bone mass, but the underlying mechanisms are poorly characterized. Using *Gfi1* knock-out mice (Gfi1-ko/ko) as SCN model, we studied the relationship between neutropenia and bone mass upon different pathogen load conditions. Our analysis reveals that Gfi1-ko/ko mice kept under strict specific pathogen free (SPF) conditions demonstrate normal bone mass and survival. However, Gfi1-ko/ko mice with early (nonSPF) or late (SPF+nonSPF) pathogen exposure develop low bone mass. Gfi1-ko/ko mice demonstrate a striking rise of systemic inflammatory markers according to elevated pathogen exposure and reduced bone mass. Elevated inflammatory cytokines include for instance Il-1b, Il-6, and Tnf-alpha that regulate osteoclast development. We conclude that low bone mass, due to low neutrophil counts, is caused by the degree of systemic inflammation promoting osteoclastogenesis.

## Introduction

Neutrophils are the predominant innate immune cell subtype that mediates initial response to infection [1]. Via chemoattractive cues neutrophils are recruited from the circulation to sites of infection and attack bacterial or fungal pathogens with an arsenal of potent antimicrobial mechanisms. These mechanisms include the release of cytotoxic molecules via degranulation of intracellular vesicles, phagocytosis, generation of neutrophil extracellular traps, production of reactive oxygen species, and synthesis of prostaglandins/leucotrienes [1, 2]. Severe congenital neutropenia (SCN) or cyclic neutropenia (CN) are immunodeficiency disorders characterized by very low neutrophil blood counts, resulting in life-threatening infections [3, 4]. Familial forms of SCN or CN are caused by mutations within the neutrophil elastase (*ELANE*), growth factor independence 1 transcription repressor (*GFI1*), HCLS1-associated protein X-1 (*HAX1*), glucose-6-phosphatase 3 (*G6PC3*), Wiskott-Aldrich syndrome protein (*WAS*), and vacuolar protein sorting 45 homolog (*VPS45*) genes [5]. Congenital neutropenia is most frequently caused by mutations within the *ELANE* gene [3]. Apart from hematopoietic defects, SCN or CN may also affect other organ systems, leading to additional clinical manifestations such as neurological signs (*HAX1*), hepatospleno- and nephromegaly (*VPS45)*, or complex defects of organ development (*G6PC3*) [3, 5]. A largely uncharacterized aspect of low neutrophil counts is osteopenia and osteoporosis [6–9].

Low bone mass upon development of neutropenia has originally been addressed in a genetically undefined cohort of chronic idiopathic neutropenia patients [9]. In that study two groups with either mild (1700–2500 neutrophils/μl) or pronounced (<1700/μl) neutropenia showed a significant reduction in bone mineral density (BMD) [9]. Further analysis demonstrated elevated serum levels of biochemical markers for bone formation and resorption as well as inflammatory activity. This suggests that increased bone cell activity, potentially triggered by an inflammatory environment, causes low BMD upon neutropenia [7, 9]. Another study confirmed low BMD also in SCN patients [10]. Since these patients require a treatment with synthetic granulocyte colony stimulating factor (G-CSF) to enhance neutrophil production it has been difficult to differentiate between the impact of G-CSF treatment and low neutrophil numbers on bone homeostasis. Long term G-CSF administration has been described to cause osteoporosis and bone pain in neutropenia patients [10]. Moreover, a specific correlation between human neutropenia (SCN, CN), the degree of osteopenia, and the underlying genetic defects is not available so far. None of the genes mutated in congenital neutropenia, with exception of *WAS*, have been linked with bone cell function or bone phenotypes. The *WAS* protein, however, is implicated in osteoclast sealing zone formation suggesting an impact at late differentiation stages [11].

SCN patients affected by dominant negative *GFI1* mutations demonstrate low neutrophil numbers, elevated monocytic cells, and diminished naive T lymphocytes in the peripheral blood [12]. This phenotype strongly overlaps with that of *Gfi1* knock-out mouse models (Gfi1-ko/ko) showing defects of the adaptive and innate immune system such as reduced lymphocyte numbers, neutropenia, and immature monocyte accumulation [13, 14]. In addition, Gfi1-ko/ko mice are growth retarded with a body mass reduction of approx. 50% [13]. Serum levels of inflammatory mediators such as Tnf-alpha, Il-10, and Il-1beta are elevated [14]. Thus, Gfi1 promotes proliferation and differentiation of lymphocytes and neutrophils, but inhibits monocyte formation [15]. Gfi1 targets diverse cell-cycle regulators, transcription factors, and granulocyte-specific genes for instance *ELA2*, *P21/WAF*, *GFI1B*, *cMyc*, *IL-2*, and *IL-6R* [16]. Recently, in a multiple myeloma (MM) model elevated expression of Gfi1 mRNA in bone marrow stromal cells and pre-osteoblasts was detected after stimulation with TNF-alpha, IL-7, or MM cell culture supernatant [17]. This study hypothesizes a role of Gfi1 in mesenchymal cell differentiation as mediator of inflammatory stimuli. Here, we utilize Gfi1-ko/ko mice as model for severe congenital neutropenia to assess the cellular and molecular mechanisms leading to low bone mass.

## Materials and methods

### Study design

Here, we study the impact of severe congenital neutropenia on bone tissue in a Gfi1 knock-out model, lacking a key transcription factor of neutrophil differentiation. The overall study design was a series of controlled laboratory experiments in mice as described below. Our main objective was to assess the effect of neutropenia in combination with different housing conditions of variable pathogen load, on bone tissue development during early adulthood of Gfi1-ko/ko mice.

The personnel performing the *in vitro* assays, microCT, and histological analysis were blinded for group allocation. Sample sizes were selected to minimize the number of animals needed while obtaining a statistically significant result. No samples or data were excluded from the data analysis. If not otherwise indicated values of Gfi1-ko/ko mice were calculated vs. Gfi1-wt/wt mice of identical housing conditions and age.

### Mouse breeding and genotyping

Gfi1-ko/ko mice, generated by Karsunky and colleagues, were bred under different conditions and genotyped as described previously (Panel A in S1 Fig) [14]. Mice bred under nonSPF conditions were born and kept in facility 1 until euthanization. Mice bred under SPF conditions were born and kept in facility 2 until euthanization. Mice bred under SPF+nonSPF conditions were born and grew up in facility 2 until 6 weeks and kept in facility 3 until euthanization. Gfi1-wt/wt and Gfi1-ko/ko mice were strictly bred in a C57Bl/6J background. All mice originate from the same breeding stock. To estimate pathogen load please consider summarized animal health certificates from indicated breeding facilities (S1 Table). The pathogen load was assessed with sentinels of the strains Crl:CD1(ICR) and C57BL6/J every 3 month or annually (see S1 Table). Animals were sacrificed by isoflurane anesthesia and subsequent decapitation. All experimental procedures were approved by the ‘Landesamt für Gesundheitsschutz und Technische Sicherheit (LaGeTSi), Berlin, Germany. All animal experimental procedures were carried out in accordance with the approved guidelines of the LaGeTSi.

### Histology and histomorphometry

For histological analysis, adult limbs of male mice were embedded in methyl methacrylate (MMA) according to standard laboratory procedures [18]. For histological assessment, 5μm plastic sections (RM2255, Leica, Germany) were stained with a combined von Kossa/ Toluidin or von Kossa/ Kernechtrot procedure. The growth plate was analyzed from von Kossa/ Toluidin stained sections. Bright light microscopy occurred with an Olympus BX60 (Olympus, Japan). Histomorphometric analysis of bone parameters were carried out using the Osteo-Measure histomorphometry system (Osteometrics, Atlanta,GA, USA) according to the guidelines of the American Society for Bone and Mineral Research. Nomenclature was used according ASBMR suggestions.

### Immunohistology

Immunodetection of proteins within mineralized bone tissue was achieved according to a standard procedure. Briefly, frozen tissue was sectioned (5μm) on a cryostat (Leica CM3050 S, Wetzlar, Germany). Subsequently, sections were fixed, permeabilized and antibodies were incubated in PBS buffered 3% BSA. The following antibodies were used for immunodetection: osteocalcin (ALX-210-333-C100, Enzo, USA) and anti-mouseAlexa555 (A31570, Invitrogen, USA). Sections were analyzed with epifluorescence microscopy Olympus BX60 (Olympus, Japan).

### Micro computed tomography (microCT) analysis

Bone samples (vertebra) were analyzed with low (6 μm voxel) resolution with a microCT from Skyscan 1172 (Skyscan, Belgium) [19]. For volumetric trabecular bone analysis samples were measured with a multi-sample holder at 6μm pixel size, 180° degree rotation, rotation angle 0.5°, filter Al 0.5mm, averaging 3, random movement 3, voltage 80kV, and current 120μA. Vertebrae of nonSPF breeding were analyzed by a Scanco VivaCT40 as described elsewhere [18, 20]. Nomenclature was used according ASBMR suggestions [21].

### Biochemical analysis of murine blood parameter

Plasma was collected after decapitation with standard protocols utilizing Li-Heparin capillary tubes (GK 150 200μl Gel grün, Kabe, Germany). Biochemical analysis of bone parameters was achieved with enzyme linked immunoassays detecting osteocalcin, Rankl, Opg, and rat-laps (CTX-I). Analysis was performed according to manufacture instructions. Cytokine profiles were generated using the quantitative Q-Plex^™^ (16-Plex) Mouse Cytokine ELISA (Tebu-Bio, Offenbach, Germany) according to manufactures protocol.

### Expression analysis of mRNA by quantitative PCR

Cortical bone was mechanically cleaned from adjacent muscle and connective tissue and the bone marrow was removed by centrifugation. Separated cortical bone tissue was mechanically crashed after liquid nitrogen incubation and mRNA lysates were generated with Trizol reagent (Invitrogen, Carlsbad, USA). For mRNA expression analysis, full RNA was isolated according to a laboratory standard protocol and 1μg RNA was transcribed into cDNA with RevertAid H Minus First Strand cDNA synthesis kit and random hexamer primers (Fermentas, K1632) [20, 22]. Expression was measured with a Taqman 7500 (ABI, USA) using Gapdh as endogenous control. Relative mRNA expression was quantified using the comparative Ct method. Primers of this study are available on request.

### Analysis of peripheral blood and bone marrow smears

Immediately after euthanization peripheral blood and bone marrow was supplemented with EDTA to prevent coagulation. Subsequently, cells were distributed on coated glass slides and air dried. Blood and bone marrow cell counts were performed according to standard protocols [13, 14, 23]. Briefly, bone marrow smears were stained according to May-Grünwald-Giemsa (MGG Quick Stain, Bio-Optica, Germany) and mounted (Bio Mount, Bio-Optica, Germany). Quantitative analysis occurred with 1000-times magnification. For each animal 1000 cells were differentially counted.

### Quantification of mesenchymal progenitor

For analysis of mesenchymal progenitor cells (MPCs), bone marrow was removed from murine tibia and femur as described earlier [24, 25]. Briefly, dissected bones were cut open with scissor, transferred into a microtube and the bone marrow was flushed out by centrifugation (500xg for 1 min). Bone marrow was weighed and resuspended in 5ml pre-warmed culture media (DMEM containing 10% FBS, 1% Pen-Strep, 1% Glutamax). The single cell suspension was seeded onto a 6-well microplate (1ml/well [CFU Assay; 2 wells/animal]; 2ml/well [RNA isolation; 1 well/animal]), incubated at 37°C and 5% CO2 for 72h and medium was changed every 2–3 days. To determine the CFU ability in each animal group, cells were fixed using 4% paraformaldehyde and stained using an alkaline phosphatase stain kit (Vector Blue APS Kit III, Vector Laboratories) and 0.1% nuclear fast red-aluminum sulfate solution as counterstaining. Total and alkaline phosphatase positive colonies were counted by microscopic imaging and quantitative image analysis (AxioVision, Zeiss, Germany).

Osteogenic differentiation of confluent bone marrow-derived mesenchymal progenitor cells was induced with osteogenic media as described previously [25]. Briefly, matrix mineralization was visualized with Alizarin Red staining. Quantification was achieved by measuring the absorbance of Alizarin Red (ODAR) after extraction with 10% cetylpyridinium chloride. Obtained values were normalized to number of viable cells determined by alamarBlue (Invitrogen).

### Statistical analysis

The IBM^®^ SPSS^®^ 22.0 software package (IBM Corp., Chicago, IL, USA) and Microsoft Excel was used for statistical evaluation. Results are presented as mean ± standard deviation (S. D.). Statistical significance between Gfi1-ko/ko mice and Gfi1-wt/wt mice (of identical housing conditions and age) was calculated using the unpaired two-sided Student’s t-test (* p ≤ 0.05, ** p ≤ 0.01). Detailed information about error bars, sample sizes, and number of technical replicates are included in all figure legends. If not otherwise indicated values of Gfi1-ko/ko mice were calculated vs. Gfi1-wt/wt mice of identical housing conditions and age.

## Results

### Housing conditions determine development of Gfi1-ko/ko mice

Gfi1 is a transcriptional regulator required for lymphoid, myeloid, and erythroid lineage development [26–29]. Gfi1-ko/ko mice are severely growth retarded and die prematurely [13, 14]. To test the impact of housing conditions on development, we kept Gfi1-ko/ko and corresponding Gfi1-wt/wt mice in three colonies—specific pathogen free (SPF), nonSPF, and SPF+nonSPF—with variable degree and timing of pathogen load (S1 Table). Compared to the SPF facility, bacterial germs (*Pasteurellaceae*, *Pasteurella pneumotropica*, *Heliobacter spp*.*)* and parasites (*Trichomonas sp*. and *Flagella*) were present in the nonSPF and SPF+nonSPF facility (S1 Table). These germs are common in mice breeding areas [30]. Mice at nonSPF were grown for 6 weeks, as Gfi1-ko/ko mice frequently die later this time point [13, 14]. Mice at SPF were bred for 8 weeks, as at this time point growth and bone development is complete. Mice at SPF+nonSPF were grown for 6 weeks in SPF followed by 4 weeks at nonSPF conditions as this ensures normal survival/initial development and is sufficient to impact bone mass due to the pathogenic environment. We analyzed offspring that were kept at indicated conditions.

**Fig 1:**
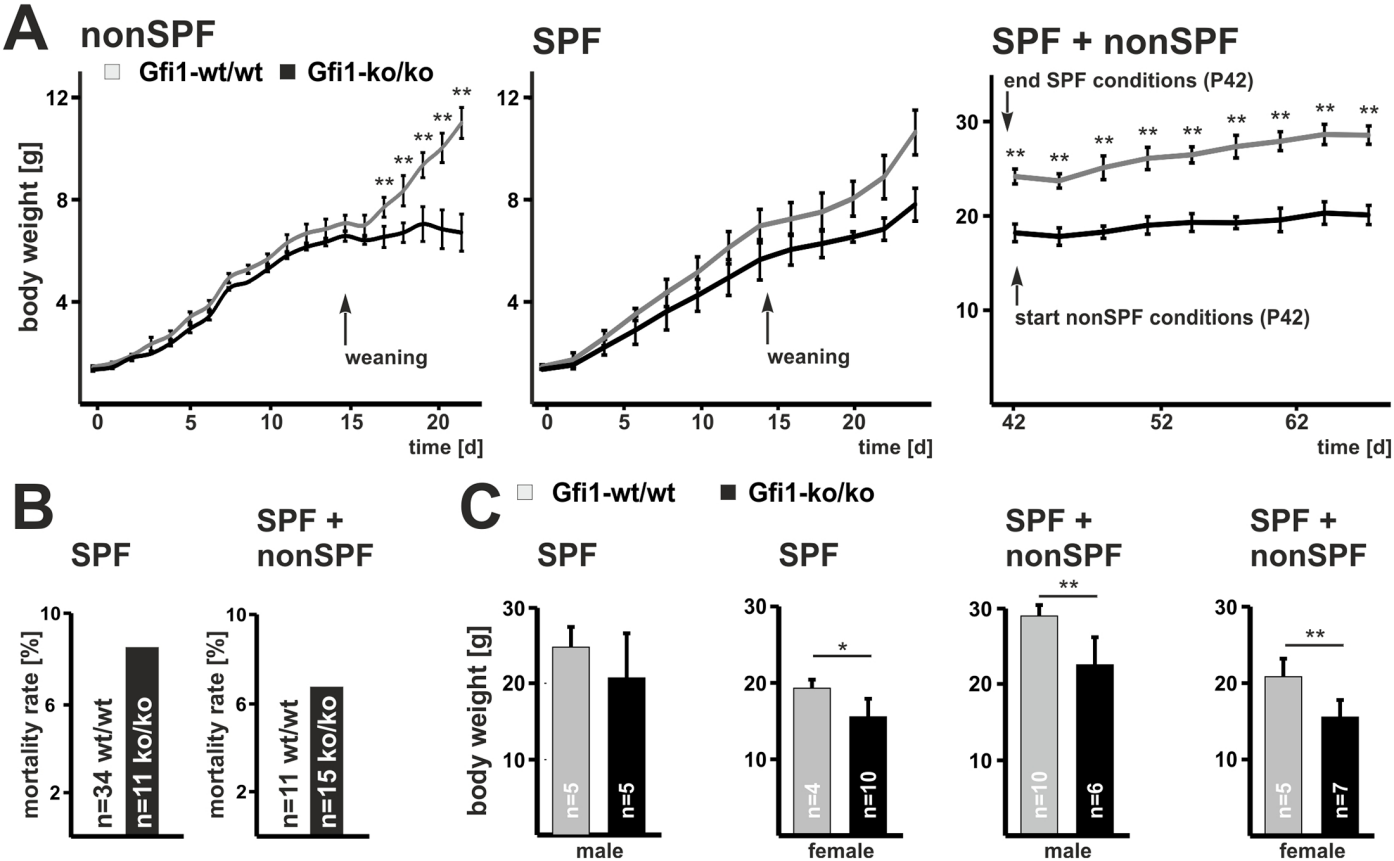
Housing conditions determine Gfi1-ko/ko mice body mass and survival. **(A)** Average growth curves from control mice kept under nonSPF conditions indicate evolving development also beyond weaning (Gfi1-wt/wt n = 4, Gfi1-ko/ko n = 3). However, soon after weaning (P19) Gfi1-ko/ko mutants display significantly delayed growth. Development under SPF growth conditions did not affect growth of Gfi1-ko/ko mice (Gfi1-wt/wt n = 4, Gfi1-ko/ko n = 3). Upon SPF+nonSPF housing Gfi1-ko/ko mice demonstrated relative normal growth compared to controls (Gfi1-wt/wt n = 4, Gfi1-ko/ko n = 3); however, Gfi1-ko/ko mice show significant body mass reduction. All curves show values of male mice. See S1 Table for health monitoring. **(B)** At SPF and SPF+nonSPF conditions Gfi1-ko/ko mice showed a mortality rate of approx. 9% and 6%, respectively. **(C)** Final body mass of controls and Gfi1-ko/ko mice was assessed at indicated time points for SPF and SPF+nonSPF conditions. Combined SPF+nonSPF breeding caused a body mass reduction of approx. 25% in male and female Gfi1-ko/ko mice. Error bars represent SD and statistical significance was calculated with t-test, * p ≤ 0.05 and ** p ≤ 0.01.

Until weaning nonSPF Gfi1-ko/ko mice show a body mass development comparable to the wild-type controls (Fig 1A). However, after weaning, assigning the switch between maternal and own immunity, nonSPF Gfi1-ko/ko mice almost stopped gaining weight. In accordance to earlier studies a large proportion of nonSPF Gfi1-ko/ko mice die at about 5–6 weeks of age [13, 14]. In contrast, Gfi1-ko/ko mice kept under SPF conditions show a mortality rate of below 10% (Fig 1B). Female and male SPF Gfi1-ko/ko mice do not show growth retardation after weaning (Fig 1A, S2 Fig). Similarly, Gfi1-ko/ko mice that first develop under SPF conditions and were later transferred into a nonSPF unit (SPF+nonSPF) do not demonstrate enhanced mortality rates, although their final body weights are reduced compared to the corresponding Gfi1-wt/wt controls (Fig 1C). After six weeks of age, nonSPF Gfi1-ko/ko mice exhibit a reduced final body weight of about 50% compared to Gfi1-wt/wt mice [13, 14]. The final body weight of SPF and SPF+nonSPF Gfi1-ko/ko mice is also reduced but to a lesser extent. Altogether, these observations suggest that pathogen exposure critically controls growth of Gfi1-ko/ko mice.

### Gfi1-ko/ko mice develop low bone mass depending on the specific pathogen load

**Fig 2:**
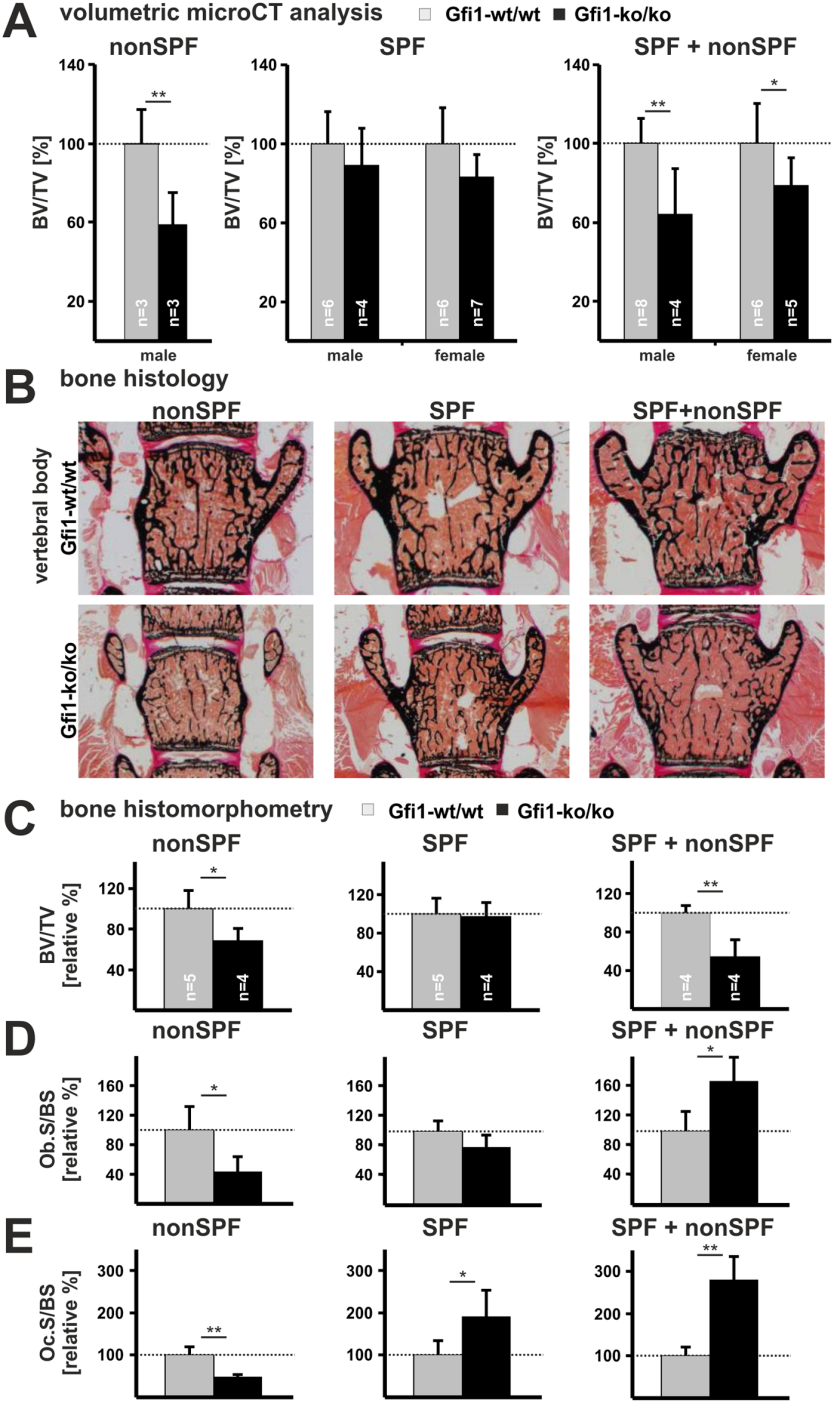
Gfi1-ko/ko mice kept under nonSPF conditions develop osteopenia. **(A)** Trabecular bone of vertebrae was measured with microCT. Gfi1-ko/ko mice kept under nonSPF conditions developed severe osteopenia as indicated by 60% BV/TV reduction compared to Gfi1-wt/wt mice. Upon SPF breeding Gfi1-ko/ko mice show a 15% reduction in BV/TV. However, SPF+nonSPF conditions induced intermediate osteopenia characterized by approx. 30% BV/TV reduction. Please note Gfi1-ko/ko mice kept at nonSPF conditions develop cortical bone osteopenia in femur. **(B)** Representative bone sections stained with von Kossa/ Kernechtrot illustrate trabecular bone in vertebrae of mice kept under nonSPF, SPF and SPF+nonSPF conditions. **(C)** Quantification of bone tissue by histomorphometry revealed reduced BV/TV values in Gfi1-ko/ko vertebrae under nonSPF and SPF+nonSPF conditions compared to controls. Breeding within the SPF environment did not significantly affect bone mass of Gfi1-ko/ko mice. **(D)** Histomorphometric quantification of the osteoblast covered bone surface (Ob.S/BS) shows reduced but elevated values upon nonSPF and SPF+nonSPF breeding, respectively. SPF breeding did not affect Ob.S/BS between control and mutant mice. **(E)** Quantification of the osteoclast covered bone surface (Oc.S/BS) revealed diminished but raised values in Gfi1-ko/ko mice upon nonSPF and SPF+nonSPF breeding compared to their corresponding Gfi1-wt/wt controls, respectively. Gfi1-ko/ko mutants grown under SPF conditions showed elevated Oc.S/BS counts compared to Gfi1-wt/wt mice. For better comparability, Gfi1-wt/wt values were set to 1 and Gfi1-ko/ko values were relatively calculated for each breeding condition. Please see S2 and S3 Tables for absolute values. Error bars represent SD and statistical significance was calculated with t-test, * p ≤ 0.05 and ** p ≤ 0.01.

Human SCN is associated with low bone mass [6, 8]. To test a role of Gfi1 in bone development, we first analyzed vertebra from nonSPF mice by microCT. The Gfi1-ko/ko mice under nonSPF conditions demonstrate 60% lower trabecular bone volume/ total volume (BV/TV) compared to their Gfi1-wt/wt controls (Fig 2A, S2 Table). In contrast, vertebrae of Gfi1-ko/ko mice housed under SPF conditions do not show a statistically significant BV/TV reduction compared to their corresponding wild-type control. Gfi1-ko/ko mice maintained under SPF+nonSPF conditions develop an intermediate phenotype characterized by approx. 30–40% diminished BV/TV relative to the controls. Other bone parameters such as trabecular number (Tb.N) and thickness (Tb.Th) are consistently reduced in nonSPF and SPF+nonSPF Gfi1-ko/ko mice but not in SPF Gfi1-ko/ko mice (except of the Tb.Th) compared to their appropriated controls (for absolute values see S2 Table).

Histological evaluation of von Kossa/ Kernechtrot stained calcified vertebra sections confirm low bone mass in nonSPF and SPF+nonSPF Gfi1-ko/ko mice as detected by micro-CT analysis (Fig 2B). Histomorphometry demonstrates a significant BV/TV reduction in nonSPF and SPF+nonSPF vertebra, while SPF mice show unaffected BV/TV compared with the corresponding controls (Fig 2C, for absolute values see S3 Table). Diminished BV/TV in nonSPF and SPF+nonSPF Gfi1-ko/ko mice compared to controls are associated with reduced Tb.Th and Tb.N. Low bone mass is not due to developmental growth plate abnormalities but diminished primary spongiosa is already visible at P14 in nonSPF Gfi1-ko/ko femur (S3 Fig). Gfi1-ko/ko mice at SPF or nonSPF conditions did not show abnormal teeth development. This suggests that high pathogen load induces low bone mass in Gfi1-ko/ko mice.

### Timing of pathogen exposure determines osteoblast and osteoclast numbers in Gfi1-ko/ko bone tissue

To assess the cellular origin of osteopenia after *Gfi1* ablation, we determined the numbers of osteoblasts and osteoclasts by histomorphometry. This reveals a significant reduction of bone forming osteoblasts in nonSPF Gfi1-ko/ko mice by more than 50% relative to their wild-type controls (Fig 2D, S3 Table). In contrast, under SPF+nonSPF conditions the N.Ob/B.Pm and Ob.S/BS are significantly increased by approx. 40% in Gfi1-ko/ko mice compared to wild-type mice. SPF Gfi1-ko/ko mice demonstrate no significant alterations of N.Ob/B.Pm and Ob.S/BS. Bone resorbing osteoclasts are significantly diminished by approx. 50% in nonSPF Gfi1-ko/ko mice compared to controls (Fig 2E, S3 Table). SPF+nonSPF Gfi1-ko/ko mice, however, exhibit significantly elevated numbers of osteoclasts. Breeding under SPF conditions significantly increases the number of osteoclasts in Gfi1-ko/ko mice compared to controls. In summary, depending on the time point of pathogen exposure low bone mass in Gfi1-ko/ko mice can be either a consequence of low or high bone cell activity.

### Low bone cell activity causes osteoporosis in nonSPF Gfi1-ko/ko mice

**Fig 3:**
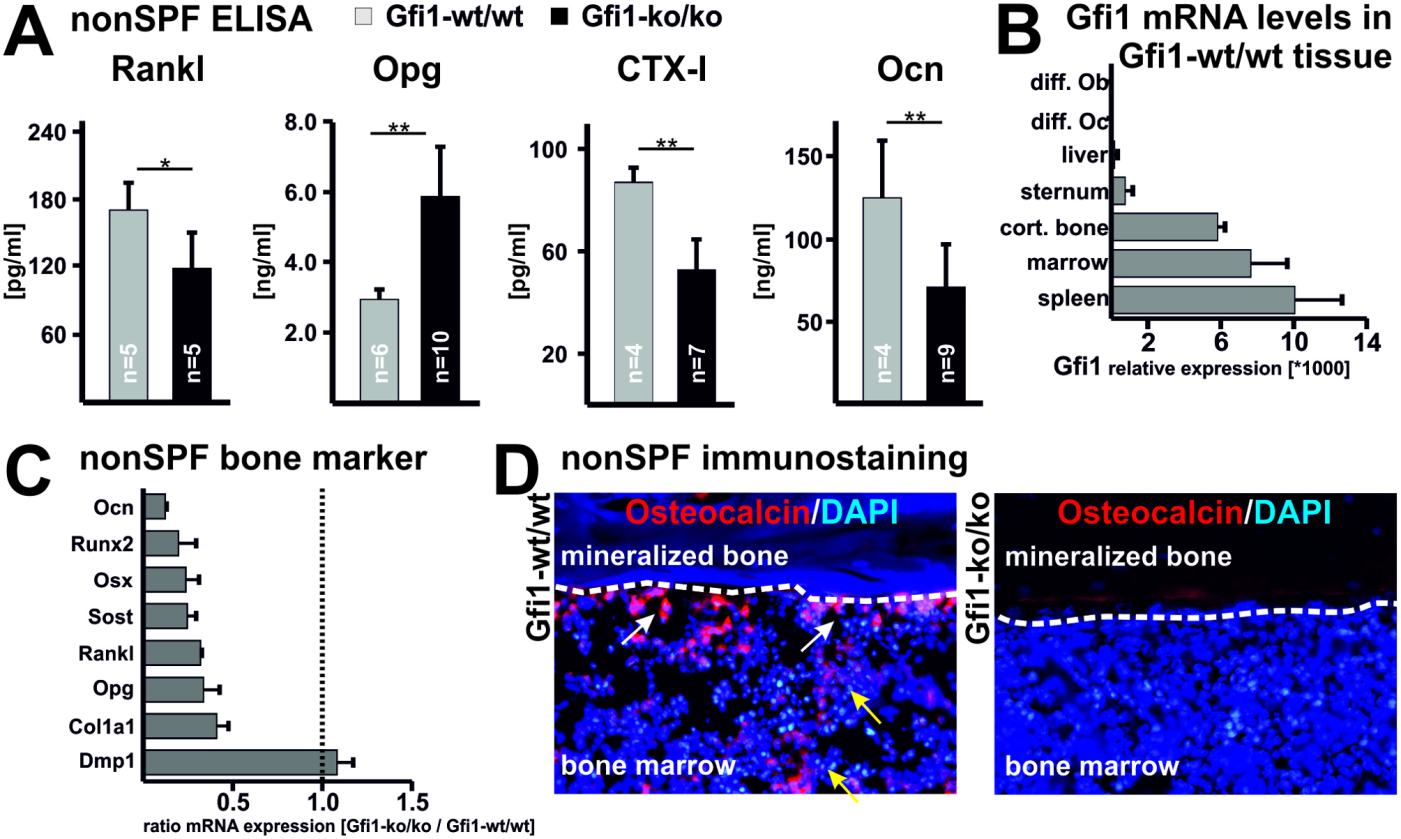
Diminished osteoblast and osteoclast activity in nonSPF Gfi1-ko/ko mice. **(A)** The Ob. and Oc. activity in Gfi1-ko/ko and Gfi1-wt/wt mice kept at nonSPF conditions was determined by measuring the plasma markers receptor activator of NF-kB ligand (Rankl), osteoprogerin (Opg), cross-linked carboxy-terminal telopeptide of type I collagen (CTX-I), and osteocalcin. Elevated Opg and diminished Rankl levels in Gfi1-ko/ko mutants suggest reduced osteoclast activity. Diminished bone resorption and formation in Gfi1-ko/ko mice is indicated by lowered CTX-I and osteocalcin levels, respectively. Error bars represent SD and statistical significance was calculated with t-test, * p ≤ 0.05 and ** p ≤ 0.01. **(B)** Basal expression of Gfi1 mRNA in different tissues shows highest values in spleen, bone marrow, and cortical bone. Non-haematopoietic tissues such as liver or cartilage and *in vitro* differentiated Oc. as well as Ob. demonstrated very low Gfi1 mRNA expression (n = 3 with 3 technical replicates/sample). Gfi1 expression was assessed with quantitative PCR (qPCR) and Gapdh was used as endogenous control. **(C)** All markers of Ob. proliferation and differentiation are diminished in Gfi1-ko/ko mutant lysates. Please note the low levels of osteoblast markers Ocn, Runx2, and Osx but normal expression of the osteocyte marker Dmp1. Values are shown as ratio of Gfi1-ko/ko vs. Gfi1-wt/wt mRNA expression (n = 3 with 3 technical replicates/sample). Normal expression is indicated with the dotted line at 1. Relative expression of bone marker genes was assessed by qPCR in full RNA preparations of cortical bone. Gapdh was used as endogenous control. **(D)** Immunohistology of the Ob. marker osteocalcin (Bglap) demonstrates abundant signals (red) in controls at the bone marrow to bone junction but low levels in Gfi1-ko/ko mutants. Please note abundant osteocalcin signals also in bone marrow cells. Immunostaining was performed on film supported cryosections. Counterstaining occurred with DAPI visualizing cell nuclei.

To investigate the molecular mechanisms behind the altered bone homeostasis we measured serum markers of bone cell function by ELISA. Systemic levels of receptor activator of NF-kB ligand (Rankl), which promotes osteoclast formation, are significantly lower in nonSPF Gfi1-ko/ko mice compared to their corresponding wild-type controls (Fig 3A). In contrast, the Rankl decoy receptor osteoprotegerin (Opg), which is expressed by osteoblasts and stromal cells, is significantly elevated by approx. 50% in nonSPF Gfi1-ko/ko mice. The bone resorption marker carboxy-terminal collagen crosslinks (CTX-I) and the formation marker osteocalcin are both significantly diminished in nonSPF Gfi1-ko/ko mice. These data corroborate a low bone cell activity situation in nonSPF Gfi1-ko/ko mice in relation to the wild-type animals.

We next analyzed the basal Gfi1 expression level in different cells and tissues from wild-type mice. Liver, sternum cartilage, and mature *in vitro* differentiated osteoclasts or osteoblasts show almost absent Gfi1 expression (Fig 3B). Consistently, *Gfi1* expression has not been identified in osteocytes that terminally differentiate from osteoblasts [31]. However, in hematopoietic organs of wild-type animals, such as bone marrow and spleen, Gfi1 is abundantly expressed. Notably, cortical bone (bone marrow was removed by centrifugation) exhibits high Gfi1 expression. This could be due to mesenchymal and hematopoietic progenitor cells localizing to the bone surface (stem cell niche). Expression of osteoblast markers in nonSPF Gfi1-ko/ko cortical bone reveals a consistent reduction of all genes tested, e.g., osteocalcin (Ocn), Runx2, osterix (Osx), sclerostin (Sost), Rankl, Opg, and collagen 1 a1 (Col1a1) (Fig 3C). The mRNA level of the osteocyte marker dentin matrix protein 1 (Dmp1) is unaffected. In accordance with diminished mRNA levels, the osteocalcin protein is expressed to a much lower extent at the bone marrow-bone-junction of nonSPF Gfi1 mice compared with the wild-type controls (Fig 3D). Overall, these findings indicate that nonSPF Gfi1-ko/ko mice have low bone cell activity due to restrained osteoblast and osteoclast development.

### Osteopenia in SPF+nonSPF Gfi1-ko/ko mice is due to increased bone cell activity

**Fig 4:**
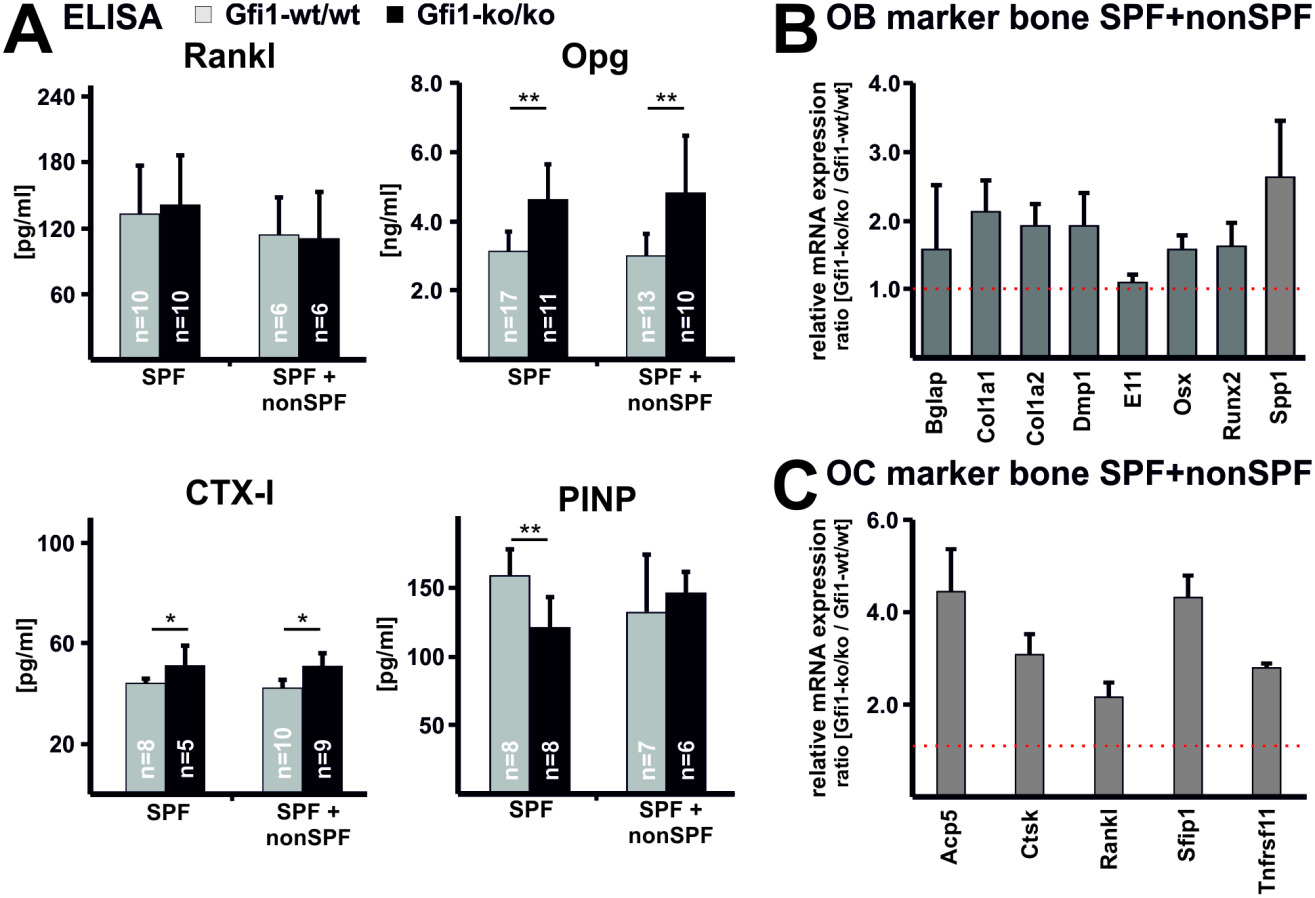
Gfi1-ko/ko mutants upon SPF+nonSPF housing show elevated osteoclast activity and bone cell marker expression. **(A)** In Gfi1-ko/ko mutant mice kept under SPF and SPF+nonSPF Rankl plasma levels are unaffected and Opg is elevated compared to Gfi1-wt/wt mice. Bone resorption (CTX-I) is mildly increased in Gfi1-ko/ko mice under both conditions. Bone formation (PINP) is mildly reduced in SPF Gfi1-ko/ko mice compared to Gfi1-wt/wt but not under SPF+nonSPF conditions. Error bars represent SD and statistical significance was calculated with t-test, * p ≤ 0.05 and ** p ≤ 0.01. **(B)** Relative expression of bone marker mRNA was analyzed with qPCR and Gapdh was used as endogenous control. Results are presented as ratio Gfi1-ko/ko vs. Gfi1-wt/wt (n = 3 with 3 technical replicates/sample; error bar indicates SD). Normal expression is indicated with the dotted line at 1. All Ob. specific markers such as Col1a1, Runx2, and Spp1 are approx. 2-fold elevated. **(C)** Markers indicating mature Oc. such as Acp5 and Ctsk are approx. 4-fold increased. Interestingly, the marker for Oc. progenitor cells Sfip1 (Pu.1) is also 4-fold elevated. Relative expression of Oc. marker mRNA was analyzed with qPCR and Gapdh was used as endogenous control.

Gfi1-ko/ko mutant mice kept under nonSPF or SPF+nonSPF conditions both develop a low bone mass phenotype (Fig 2A–2C). In contrast to nonSPF Gfi1-ko/ko mice, SPF+nonSPF Gfi1-ko/ko mutants show increased osteoblast and osteoclast numbers compared to the corresponding wild-type controls (Fig 2D and 2E, S3 Table). Serum concentration of Rankl appears unaffected (Fig 4A); while Opg and CTX-I levels are mildly increased in SPF and SPF+nonSPF Gfi1-ko/ko mice compared to the corresponding wild type controls. Serum PINP concentrations are diminished in SPF but normal in SPF+nonSPF Gfi1-ko/ko mice compared to Gif1-wt/wt mice. Consistent with increased osteoblast numbers, mRNA levels of osteoblast specific genes such as Col1a1, Osx, Runx2, and Spp1 are elevated in SPF+nonSPF Gfi1-ko/ko mice relative to the controls (Fig 4B). Similarly, elevated expression of osteoclast markers, e.g., tartrate-resistant acid phosphatase (Acp5), cathepsin K (Ctsk), and the Rank receptor (Tnfrsf11) correlates well with increased osteoclast numbers (Fig 4C). A similar trend in mRNA expression of Ob. and Oc. marker genes was observed for Gfi1-ko/ko mice kept at SPF conditions only (S4 Fig). Notably, the relative increase in osteoblast and osteoclast numbers in SPF+nonSPF Gfi1-ko/ko mice is approx. 1.5-fold and 3-fold, respectively. Thus, SPF+nonSPF Gfi1-ko/ko mice develop osteopenia due to increase bone cell activity that likely favors bone resorption by osteoclasts. Normal osteoblast and 2-fold elevated osteoclast numbers in SPF Gfi1-ko/ko mice support this idea.

Slightly diminished osteoblast numbers in nonSPF mice could indicate that Gfi1 regulates the mesenchymal progenitor pool. In order to test an endogenous impact of Gfi1 on osteoblast progenitor cell number and differentiation we performed *in vitro* culture of bone stromal cells derived from nonSPF animals. *In vitro* cultured bone stromal cells from Gfi1-wt/wt controls were analyzed for the expression of marker genes. Gfi1 mRNA shows high expression that is comparable with the levels of stromal cell markers nestin (Nes), alkaline phosphatase 2 (Akp2), Cd73, and Cd90 (Panel A in S5 Fig). Consistently, high levels of pre-osteoblast and osteoblast markers such as Col1a1, osteopontin (Spp1), and Opg suggest appropriate technical execution. To test a role of Gfi1 in mesenchymal progenitors, we analyzed their quantity as alkaline phosphatase positive colony forming units (CFU-ALP) Gfi1-ko/ko and Gfi1-wt/wt mice kept under SPF conditions. We choose SPF mice as these animals develop no severe overall and bone phenotype. Image analysis demonstrate reduced CFU-ALP colony number, diminished CFU-ALP colony area, and decreased number of ALP positive cells per colony in Gfi1-ko/ko mice relative to the controls (Panel B in S5 Fig). Moreover, matrix mineralization of SPF *in vitro* differentiated MSCs demonstrated a significant reduction in Gfi1-ko/ko cells compared to controls (Panel C in S5 Fig). These finding suggest a role of Gfi1 in the maintenance, proliferation, and/or differentiation of mesenchymal progenitor cells.

### Defects in blood cell differentiation of Gfi1-ko/ko mice are not rescued upon SPF breeding

In order to exclude that the hitherto described alterations in bone tissue are a direct consequence to pathogen induced differences in blood cell production, we analyzed immune cell counts in peripheral blood and bone marrow. SPF and SPF+nonSPF Gfi1-ko/ko mice develop elevated neutrophil promyelocytes but diminished myelo-/metamyelocytes as well as mature neutrophil granulocytes in bone marrow (Table 1). Consistently, the number of mature neutrophils is significantly diminished in peripheral blood compared to wild-type animals (S4 Table). This is well consistent with the specific role of Gfi1 in neutrophil development [13, 14]. Gfi1-ko/ko mice further display in bone marrow normal eosinophil differentiation, unaffected monocyte numbers, lymphocytopenia, erythrocytopenia, and massive accumulation of atypical myeloid cells. Concordantly, expression of the lymphocyte marker genes Cd3e, Cd4, as well as Cd8a, and the neutrophil marker Ly6g is reduced in bone marrow (Panels A-B in S6 Fig). The observation of erythropenia in Gfi1-ko/ko mice at SPF and SPF+nonSPF conditions supports an impact of Gfi1 for erythroid lineage development [26, 28]. Compared to SPF conditions only the lymphocytopenia is more pronounced in SPF+nonSPF Gfi1-ko/ko mice (Table 1). Thus, increased pathogen contact negatively influences lymphocyte production in Gfi1-ko/ko mutants.

**Table 1:** Differential bone marrow cell count of Gfi1 mice kept under SPF and SPF+nonSPF conditions.

		SPF conditions			SPF+nonSPF conditions			SPF vs. SPF+nonSPF	
value	unit	control	Gfi1 hom	t-test	control	Gfi1 hom	t-test	t-test controls	t-test Gfi1 hom
n		4	3		3	4			
**neutrophile promyelocytes**	%	1.75 ± 0.66	4.90 ± 1.14	p ≤ 0.01	2.63 ± 0.75	5.18 ± 1.32	p ≤ 0.05	n.s.	n.s.
**neutrophile myelo- and metamyelocytes**	%	14.63 ± 4.97	0.07 ± 0.06	p ≤ 0.01	17.70 ± 11.79	0.43 ± 0.46	p ≤ 0.05	n.s.	n.s.
**neutrophile granulocytes**	%	7.33 ± 0.97	0.00 ± 0.00	p ≤ 0.01	9.80 ± 6.77	0.00 ± 0.00	p ≤ 0.05	n.s.	n.s.
**eosinophile promyelocytes**	%	0.30 ± 0.08	0.57 ± 0.31	n.s.	0.30 ± 0.10	0.40 ± 0.08	n.s.	n.s.	n.s.
**eosinophile myelo- and metamyelocytes**	%	2.28 ± 0.53	3.83 ± 1.47	n.s.	1.83 ± 0.78	2.45 ± 0.76	n.s.	n.s.	n.s.
**eosinophile granulocytes**	%	0.83 ± 0.26	0.40 ± 0.20	n.s.	0.90 ± 0.62	0.43 ± 0.39	n.s.	n.s.	n.s.
**lymphocytes**	%	6.40 ± 3.57	4.87 ± 1.07	n.s.	7.63 ± 2.15	2.25 ± 0.95	p ≤ 0.01	n.s.	p ≤ 0.05
**monocytes**	%	0.53 ± 0.17	0.70 ± 0.44	n.s.	0.47 ± 0.25	0.88 ± 0.46	n.s.	n.s.	n.s.
**erythrocytes**	%	65.15 ± 4.41	36.47 ± 8.38	p ≤ 0.01	58.23 ± 18.92	27.53 ± 4.76	p ≤ 0.05	n.s.	n.s.
**atypical myeloid cells (ring nucleus)**	%	0.00 ± 0.00	1.73 ± 1.16	p ≤ 0.05	0.00 ± 0.00	1.83 ± 0.81	p ≤ 0.05	n.s.	n.s.
**atypical myeloid cells (others)**	%	0.00 ± 0.00	45.63 ± 6.37	p ≤ 0.01	0.00 ± 0.00	56.83 ± 5.24	p ≤ 0.01	n.s.	n.s.
**other blasts**	%	0.83 ± 0.71	0.83 ± 0.06	n.s.	0.50 ± 0.10	0.68 ± 0.44	n.s.	n.s.	n.s.

Statistical significance calculated by unpaired t-test of controls vs. Gfi1 hom. All values are given as mean ± standard deviation. n.s.—not significant.

### Loss of Gfi1 induces elevated G-CSF & GM-CSF levels and monocyte mobilization into the periphery but no bone marrow monocytosis

**Fig 5:**
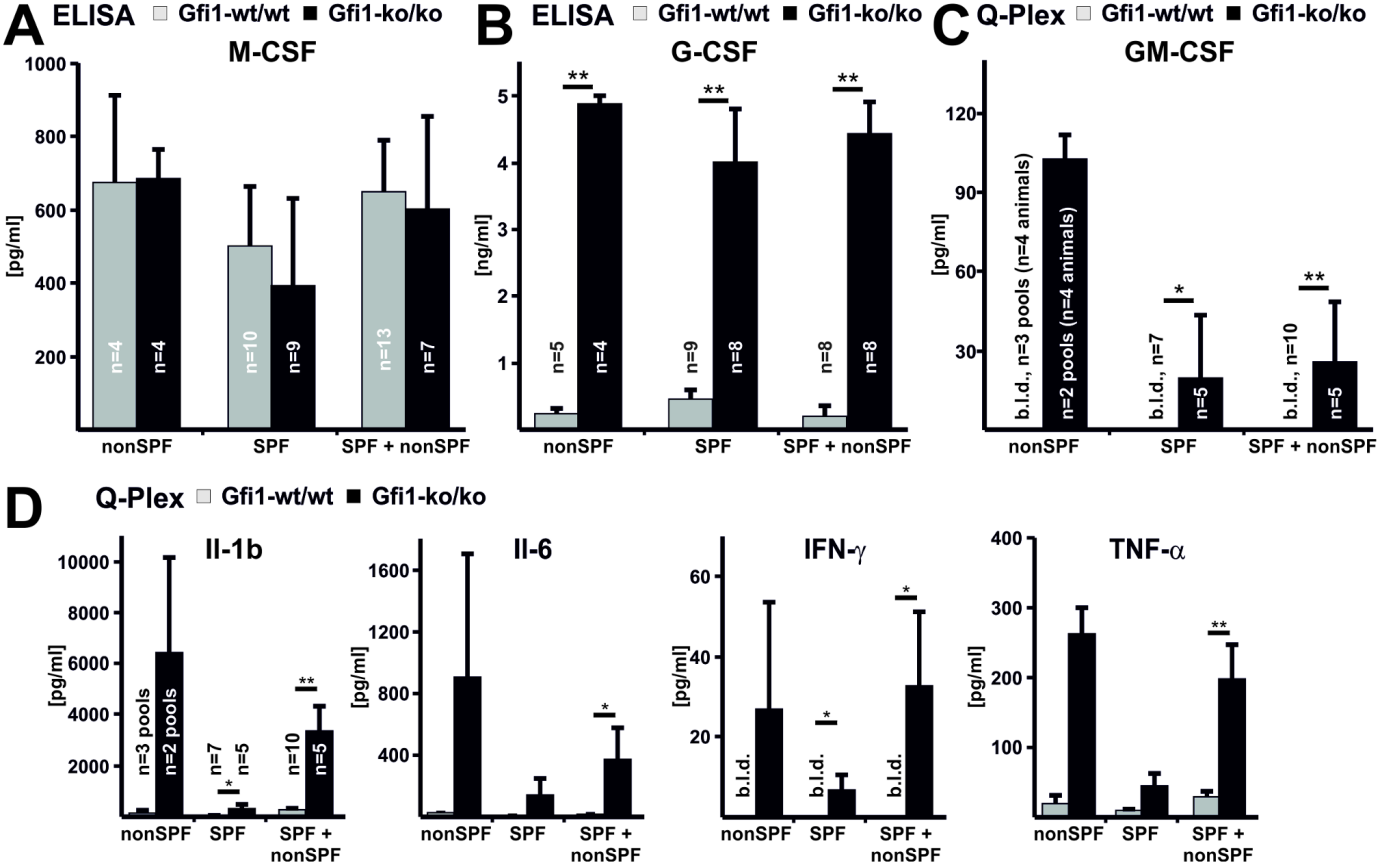
Elevated G-CSF and GM-SCF but normal M-CSF levels in Gfi1-ko/ko mice. The plasma levels of M-CSF, G-CSF, and GM-CSF were assessed by ELISA in mice kept at nonSPF, SPF, and SPF+nonSPF conditions. **(A)** M-CSF levels of Gfi1-ko/ko mutants were unaffected compared to controls upon all breeding conditions. **(B)** G-CSF is massively elevated in Gfi1-ko/ko mice compared to controls housed at nonSPF, SPF, and SPF+nonSPF conditions. **(C)** At all conditions Gfi1-ko/ko mice demonstrate significantly elevated GM-CSF levels. GM-CSF levels were measured by Q-Plex ELISA assay. The GM-CSF was below the limit of detection (b.l.d.) in Gfi1-wt/wt under all breeding conditions. Error bars represent SD. **(D)** The plasma levels of inflammatory cytokines were assessed by Q-Plex assay in control and Gfi1-ko/ko mice kept at nonSPF, SPF, and SPF+nonSPF conditions. Inflammatory cytokines such as Il-1β, Il-6, IFN-gamma, and TNF-alpha are significantly elevated in Gfi1-ko/ko mice kept at SPF+nonSPF conditions compared to their controls. Highest levels of Il-1β, Il-6, IFN-gamma, and TNF-alpha are present in Gfi1-ko/ko mice exclusively kept at nonSPF conditions. Due to sample limitations we measured for nonSPF mice serum pools (n = 2 with n = 4/pool) that were not statistically evaluated. Please note that lowest cytokine concentrations in Gfi1-ko/ko mice are present upon SPF conditions. In control mice IFN-gamma was below the limit of detection independent from the breeding condition. Full data set of analyzed cytokines is available as S5 Table. Error bars represent SEM. Statistical significance was calculated with t-test, * p ≤ 0.05 and ** p ≤ 0.01.

Regarding their monocyte production, the two independent Gfi1 knock-out models demonstrate conflicting results. While Karsunky et al. show a 7-fold elevation of monocytes at both locations, the report by Hock et al. does not identify differences in peripheral blood or bone marrow residing monocytes [13, 14]. The relative numbers of circulating monocytes are 2-fold elevated in SPF and SPF+nonSPF Gfi1-ko/ko mice compared to the corresponding controls (S4 Table). Within the bone marrow, monocytes are not significantly raised in SPF and SPF+nonSPF Gfi1-ko/ko mice (Table 1). Subsequently, we measured serum cytokine levels of macrophage colony-stimulating factor (M-CSF), granulocyte macrophage colony-stimulating factor (GM-CSF), and granulocyte colony-stimulating factor (G-CSF) of nonSPF, SPF, and SPF+nonSPF Gfi1-ko/ko mice and the corresponding controls (Fig 5A–5C). The level of M-CSF, regulating osteoclast progenitors, are unaffected in Gfi1 mice. In contrast, GM-CSF, which inhibits osteoclastogenesis by favoring macrophage differentiation [32, 33], is significantly elevated in SPF and SPF+nonSPF conditions. Also under nonSPF conditions GM-CSF is elevated in Gfi1-ko/ko mice but statistical analysis could not be performed. In addition, G-CSF, promoting granulocyte production, is strongly elevated in nonSPF, SPF, and SPF+nonSPF Gfi1-ko/ko mice. Gfi1 regulates expression of the Cxcr4 gene in myeloid cells in a G-CSF dependent manner [34]. In bone marrow lysates of SPF and SPF+nonSPF Gfi1-ko/ko mice, mRNA of Cxcr4 and its ligand Cxcl12 is diminished (Panel C in S6 Fig). Cortical bone of both mice demonstrates increased expression of Cxcl12. In summary, loss of Gfi1 stimulates monocyte mobilization into the blood stream possibly by elevated G-CSF levels [35] but does not induce marrow monocytosis. Moderate increase of monocyte levels in bone marrow might support enhanced osteoclastogenesis upon SPF+nonSPF breeding.

### Pro-inflammatory cytokines promote osteoclastogenesis in Gfi1-ko/ko mice

Due to neutropenia Gfi1-ko/ko mice expose signs of systemic inflammation [14]. Thus, we analyzed the inflammatory cytokine pattern by a Q-Plex assay in Gfi1-ko/ko mice and the wild-type controls upon all breeding conditions. Even in SPF Gfi1-ko/ko mutants show a consistent and significant up-regulation of inflammatory cytokines such as Il-1beta, Il-2, Il-3, Il-17, Mcp-1, Ifn-gamma, and Tnf-alpha compared to their wild-type counterparts (Fig 5D, S5 Table), demonstrating latent inflammation in spite of normal survival and bone development. SPF+nonSPF Gfi1-ko/ko mice show significantly increased systemic levels of most cytokines and some of them are highly elevated including Il-1b, Il-5, Il-6, or Il-12 (S5 Table). Gfi1-ko/ko nonSPF mutants develop extremely high inflammatory cytokine levels. Compared to SPF+nonSPF Gfi1-ko/ko mice, nonSPF mice demonstrate almost a doubling of Il-1b, Il-2, Il-6, Il-17, and Mcp-1 concentrations. Due to sample limitations we measured for nonSPF mice serum pools (n = 4 animals/pool, n = 2 pools) that were not statistically evaluated. Interestingly, also under SPF breeding, Gfi1-ko/ko mice show considerable elevation of inflammatory cytokines suggesting that even at very low pathogen exposure a chronic inflammation is present. Cytokines critical for osteoclast and osteoblast development such as Il-1beta, Il-6, and Tnf-alpha are consistently elevated under all breeding conditions in Gfi1-ko/ko mice relative to wild-type controls. Notably, the anti-inflammatory cytokine Il-10 is not detectable under all breeding conditions of Gfi1-ko/ko and control mice. Absence of Ifn-gamma in the control group excludes an acute T-cell mediated immune response. Thus, Gfi1-ko/ko mutants demonstrate a chronic inflammatory response in consequence to pathogen exposure.

## Discussion

Although previous reports demonstrate an association of low neutrophil counts with chronic inflammation and osteopenia in neutropenic patients [9, 10] the underlying mechanisms have been insufficiently characterized. Here, we utilize Gfi1 knock-out mice as a model for SCN to assess the mechanism leading to low bone mass. Our study reveals a critical impact of the pathogenic environment on postnatal bone development and survival of Gfi1-ko/ko mice.

### The pathogenic environment determines Gfi1 mice survival and development

**Fig 6:**
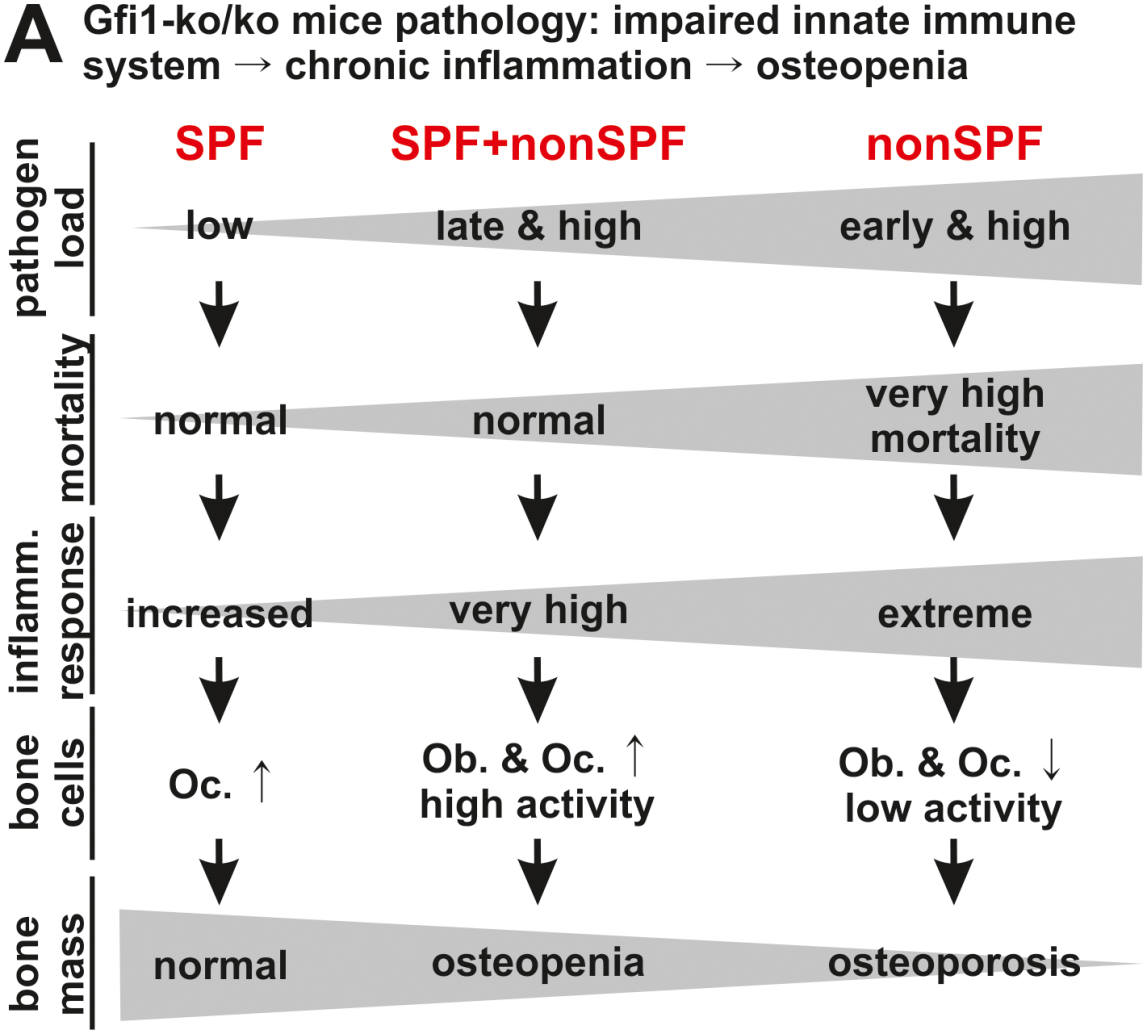
Gfi1 regulates hematopoietic and mesenchymal cells. Low bone mass in Gfi1-ko/ko mice results from inflammatory response due to severe neutropenia. Loss of Gfi1 and housing at conditions of variable pathogen load lead to increased mortality, inflammatory response, and reduction of bone mass. The inflammatory response is the main determinant of osteopenia and osteoporosis in the severe neutropenia mouse model Gfi1.

Gfi1-ko/ko mice grown under conditions of high pathogen exposure (nonSPF) exhibit growth retardation associated with osteoporosis and premature death immediately after weaning (Fig 6A). Our observation is in line with earlier studies of Gfi1 knock-out mice reporting diminished survival rates starting from two or four weeks of postnatal life [13, 14]. Normal development upon weaning suggests that maternal passive immunity via milk-derived secretory IgA at least temporally compensates for the immunodeficiency due to *Gfi1* ablation [36]. Unexpectedly, Gfi1-ko/ko mice housed under strict SPF conditions display normal development, survival, and bone mass. These Gfi1-ko/ko mice show a mortality of approx. 10% at 56 days, which is a significant improvement compared to approx. 70% [14] and 55% [13] in previous studies. Both studies mention infections and/ or abscess formation in Gfi1-ko/ko mutants but do not specify the pathogen load and weaning schedules of their SPF facilities [13, 14]. Notably, the transfer of Gfi1-ko/ko mice grown under strict SPF conditions into a nonSPF unit (SPF+nonSPF), results in a low bone mass phenotype, but does not increase their mortality rates compared to SPF Gfi1-ko/ko mice. Our systematic study demonstrates that survival and development of Gfi1-ko/ko mice critically relies on the breeding environment. Thus, the degree, quality, and timing of the pathogen exposure has to be considered for each phenotypical aspect of Gfi1-ko/ko mice.

### The pathogen load and inflammation determines bone mass in Gfi1-ko/ko mice

Early (nonSPF) and late (SPF+nonSPF) pathogen exposure of Gfi1-ko/ko mice results in osteoporosis and osteopenia, respectively. Diminished bone mass develops if bone resorption exceeds formation and can be a result of either excessive osteoclast and/ or decreased osteoblast activity [37]. Early high pathogen load (nonSPF) causes osteoporosis in Gfi1-ko/ko mice marked by an extreme systemic inflammatory response, diminished osteoclast as well as osteoblast numbers, and low bone cell activity (Fig 6A). Consistently, the systemic level of the osteoclast promoting factor Rankl is decreased, while the concentration of its decoy receptor Opg is elevated relative to the corresponding control. Later high pathogen exposure (SPF+nonSPF) induces a very high inflammatory response, increases osteoclasts as well as osteoblasts, and leads to high bone cell activity osteopenia. Strict SPF breeding causes a chronic inflammation and slightly promoted osteoclastogenesis but does not affect bone mass significantly. Normal M-CSF levels and mild elevation of bone marrow monocytes suggest that bone loss in Gfi1-ko/ko mutants is not due to boosted myeloid precursor production [38]. Strongly elevated G-CSF and GM-CSF in Gfi1-ko/ko mutants may result from diminished consumption due to defective granulocyte differentiation and high G-CSF levels likely promote monocyte mobilization into the peripheral blood [35]. Whether or not increased peripheral blood monocytes, as observed in Gfi1-ko/ko mutants, may support osteoclastogenesis is a current matter of research [39]. Elevated systemic G-CSF levels have been linked with low bone mass due to increased osteoclast and reduced osteoblast formation [10]. However, our data show that extreme systemic G-CSF levels in SPF Gfi1-ko/ko mice only mildly induce osteoclast formation and cause no bone mass reduction. This suggests that extreme G-CSF plasma levels require a co-stimulus mediating its effect on osteoclast and osteoblast formation. This co-stimulus could be the systemic inflammation that is dependent on the timing and degree of pathogen exposure and correlates with the bone mass reduction in Gfi1-ko/ko mice.

### Neutropenia affects osteoclasts and osteoblasts via systemic inflammation

Inflammation involves the activation of innate and adaptive immune cells leading to production of inflammatory cytokines [40]. Some of these molecules including GM-CSF, Il-1beta, Il-6, Il-10, Il-12, Il-17, Mcp-1, Ifn-gamma, and Tnf-alpha interact directly or indirectly with osteoblast and osteoclast differentiation and activity [38, 41]. Several studies reported that the extent of inflammatory response correlates with the magnitude of bone loss [42, 43]. However, the cytokine pattern in Gfi1-ko/ko mice are complex and comprise increased levels of molecules that support bone resorption (Il-1α, Il-1β, Il-6, Tnf-alpha, Mcp-1), diminish bone resorption (Il-12), or have context specific functions (Il-17, Ifn-gamma, GM-CSF) on bone tissue [40]. Thus, bone mass in Gfi1-ko/ko mice results from the net outcome of diverse signaling factors but not a single molecule or pathway.

In a chronic inflammatory condition (SPF) Gfi1 neutropenia mildly favors osteoclastogenesis but only a boosted inflammation (SPF+nonSPF) efficiently stimulates osteoclast formation and activity [44, 45]. Since bone resorption and formation are tightly coupled processes elevated osteoclast numbers can also induce higher osteoblast numbers [46, 47]. Recent data suggest that osteoclast-derived molecules including Bmp2, Bmp6, Tgf-beta, Wnt10b, and sphingosine 1-phosphate positively regulate osteoblast function [48–50]. Thus, a substantial increase in osteoclasts may also raise osteoblast numbers. However, massive systemic inflammation due to early and high pathogen load (nonSPF) diminishes osteoblasts and osteoclasts suggesting that beyond a certain threshold inflammatory cytokines suppress both bone cell types. Recently, elevated levels of pro-inflammatory cytokines such as TNF-alpha or IFN-gamma were shown to block bone formation either by inhibition of osteoblast differentiation or indirectly via induction of apoptosis in mesenchymal stroma cells [17, 51–53]. Extreme systemic inflammation (nonSPF) is likely the cause of high Gfi1-ko/ko mice mortality [13, 14]. We thus speculate that extreme cytokine levels compromise bone and immune cell viability and/ or function leading to osteoporosis and reduce survival rates of nonSPF Gfi1-ko/ko mice.

Our study demonstrates a critical impact of a defective innate immune system due to neutropenia and systemic inflammation for bone development. Our findings support studies showing: (i) osteoporosis in SCN patients [9, 10], (ii) an increased neutrophil to lymphocyte ratio in age-related osteoporosis [54], and (iii) deficient neutrophil recruitment as cause of local periodontal inflammatory bone loss [55]. Findings made in Gfi1-ko/ko mice established neutrophils as critical determinants of bone tissue development and homeostasis. Prospectively, the Gfi1 knock-out model may assist further characterization of the osteo-immunological interaction, linking neutrophils and bone cells. In our hands nonSPF Gfi1-ko/ko mice were severely sick limiting due to early lethality and animal welfare issues the number of usable animals. Further insights to unravel the Gfi1-ko/ko bone phenotype and the osteo-immunological interaction of neutrophils may evolve from studying the impact of food intake, early postnatal as well as aged mice, the bone formation rate, and the inflammatory environment at different time points. To elucidate the complex interplay of immunological and mesenchymal cells, approaches involving conditional Gfi1 knock-out models are additionally required in the future.

### Is Gfi1 a regulator of the mesenchymal lineage?

Gfi1 and its homologue Gfi1b are transcriptional regulators that critically control distinct steps of hematopoietic development [15]. While Gfi1b mainly regulates erythroid and megakaryocytic cells, Gfi1 determines neutrophil, lymphocyte, and HSC production [56, 57]. We did not detect Gfi1 mRNA in differentiated osteoblasts and osteoclasts. However, D´Souza et al. showed that Gfi1 represses the transcription factor Runx2 in response to Il-7 and TNF-alpha release in multiple myeloma bone disease [17]. Runx2 is crucial for osteoblast development [58]. The finding that SPF Gfi1-ko/ko mice, exposed to very low pathogen load, demonstrate chronic elevation of inflammatory markers and osteoclasts, but normal BV/TV and osteoblast numbers, could be explained by a reduced inhibitory effect of TNF-alpha on Gfi1-deficient SSCs. Osteoblast specific overexpression of Runx2, however, leads to a high bone turnover phenotype with amplified osteoclast and osteoblast numbers [59]. These findings partially mirror our observation in SPF+nonSPF Gfi1-ko/ko mice supporting the idea of Gfi1 as a mesenchymal regulator. Thus, a possible cell autonomous impact of Gfi1 on SSCs or other mesenchymal cells would extend its function in progenitor cell regulation. From a research perspective, our findings prompt for the identification of the Gfi1-related cell autonomous impact on SSCs and/or other mesenchymal cells using tissue-specific Gfi1 knockout models.

## Supporting information

S1 Fig Genotyping of Gfi1-wt/wt, Gfi1-wt/ko, Gfi1-ko/ko mice. (DOCX)

S2 Fig Growth curves of Gfi1-wt/wt and Gfi1-ko/ko female mice. (DOCX)

S3 Fig Normal growth plate and long bone development in Gfi1-ko/ko mice. (DOCX)

S4 Fig Expression of osteoblast and osteoclast marker in bone tissue of SPF Gfi1-wt/wt and Gfi1-ko/ko mice. (DOCX)

S5 Fig Gfi1 is expressed in mesenchymal stromal cells (MSC), promotes the MSC pool in vitro and is required for osteogenic in vitro differentiation. (DOCX)

S6 Fig qPCR expression analysis of Cxcl12/Cxcr4 and immune cell marker genes. (DOCX)

S1 Table Health monitoring of mice kept under nonSPF, SPF, and SPF+nonSPF conditions. (DOCX)

S2 Table Volumetric microCT analysis of trabecular in vertebra of mice kept under nonSPF, SPF, and SPF+nonSPF conditions. (DOCX)

S3 Table Histomorphometry in vertebra of Gfi1 mice kept under nonSPF, SPF, and SPF+nonSPF conditions. (DOCX)

S4 Table Differential peripheral blood cell count of mice kept under SPF and SPF+nonSPF conditions. (DOCX)

S5 Table Quantitative multiplex analysis for immunmodulatory cytokines of mice kept under nonSPF, SPF, and SPF+nonSPF conditions. (DOCX)
