# Supplementary material for: Loss of murine Gfi1 causes neutropenia and induces osteoporosis depending on the pathogen load and systemic inflammation
Source: PLoS One. 2018 Jun 7;13(6):e0198510. doi: 10.1371/journal.pone.0198510 (PMC5991660; doi:10.1371/journal.pone.0198510)
Supplement: S1 Fig — (DOCX) [file pone.0198510.s001.docx]

**S1 Figure**

**S1 Figure. Genotyping of Gfi1-wt/wt, Gfi1-wt/ko, Gfi1-ko/ko mice.**

**(A)** Gfi1-ko/ko mice were genotyped with a PCR for the wild-type (209 bp) and knock-out (320 bp) allele. Each genotyping comprised samples, a Gfi1-wt/ko positive control, and water as negative control. The shown image represents an agarose gel that has been generated in one gel run.
