# Supplementary material for: Loss of murine Gfi1 causes neutropenia and induces osteoporosis depending on the pathogen load and systemic inflammation
Source: PLoS One. 2018 Jun 7;13(6):e0198510. doi: 10.1371/journal.pone.0198510 (PMC5991660; doi:10.1371/journal.pone.0198510)
Supplement: S2 Fig — (DOCX) [file pone.0198510.s002.docx]

**S2 Figure**


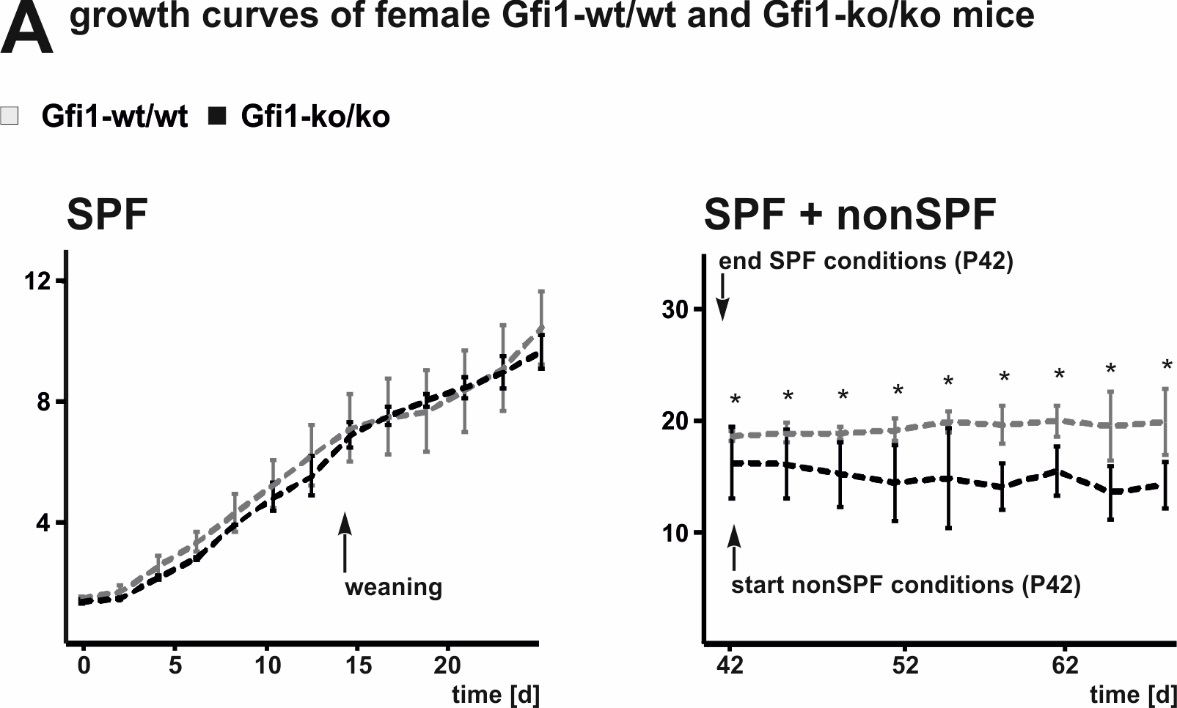


**S2 Figure. Growth curve of female Gfi1-ko/ko mice at SPF and SPF+nonSPF.**

1. Average growth curves from female control mice kept under SPF conditions indicate normal early development of both genotypes (Gfi1-wt/wt n=4, Gfi1-ko/ko n=3). Upon SPF+nonSPF housing Gfi1-ko/ko mice showed stable growth relative to controls (Gfi1-wt/wt n=3, Gfi1-ko/ko n=3). Female Gfi1-ko/ko mice show significant body mass reduction that is comparable to males (see also Fig.1). All curves show values of female mice. Error bars represent SD and statistical significance was calculated with t-test, * p ≤ 0.05.
