# Supplementary material for: Loss of murine Gfi1 causes neutropenia and induces osteoporosis depending on the pathogen load and systemic inflammation
Source: PLoS One. 2018 Jun 7;13(6):e0198510. doi: 10.1371/journal.pone.0198510 (PMC5991660; doi:10.1371/journal.pone.0198510)
Supplement: S3 Fig — (DOCX) [file pone.0198510.s003.docx]

**S3 Figure**

**S3 Figure. Normal growth plate and long bone development in Gfi1-ko/ko mice.**

**(A)** Gfi1-ko/ko mice show normal development of proliferating and differentiating growth plate cartilage. Femura were assessed at postnatal day 14 (P14). **(B)** Long bones (femur) of Gfi1-wt/wt and Gfi1-ko/ko mice were analyzed at 6 weeks and demonstrate diminished trabecular and cortical bone tissue. Bone sections were analyzed with by von Kossa/Toluidin staining and subsequent light microscopy. Femura were embedded in plastic and processed without decalcification.
