# Supplementary material for: Loss of murine Gfi1 causes neutropenia and induces osteoporosis depending on the pathogen load and systemic inflammation
Source: PLoS One. 2018 Jun 7;13(6):e0198510. doi: 10.1371/journal.pone.0198510 (PMC5991660; doi:10.1371/journal.pone.0198510)
Supplement: S4 Fig — (DOCX) [file pone.0198510.s004.docx]

**S4 Figure**


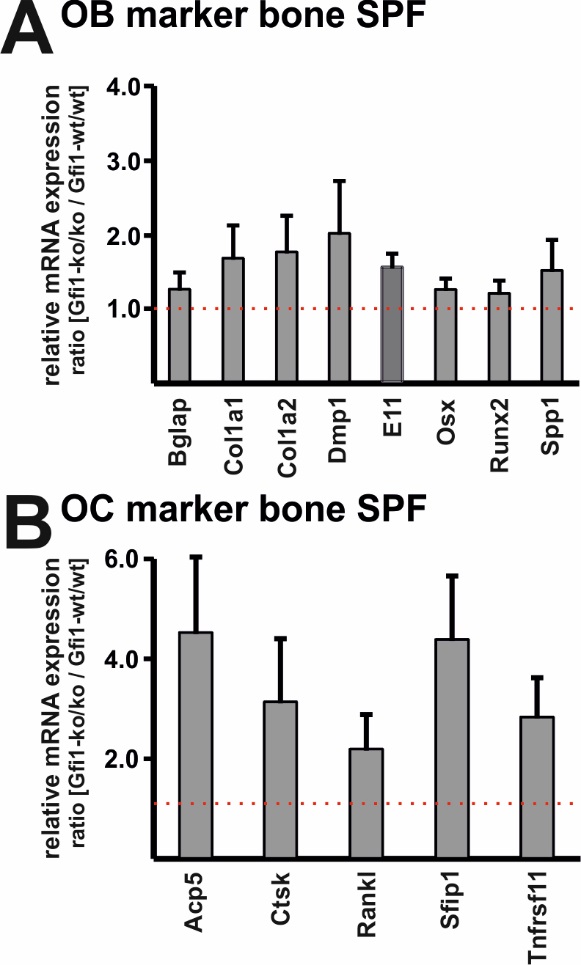


**S4 Figure. Expression of osteoblast and osteoclast marker in bone tissue of SPF Gfi1-wt/wt and Gfi1-ko/ko mice.**

**(A)** Relative expression of bone marker mRNA was analyzed with qPCR and results are presented as ratio Gfi1-ko/ko vs. Gfi1-wt/wt (n=3 with 3 technical replicates/sample; error bar indicates SD). Normal expression is indicated with the dotted line at 1. Gapdh expression was used as endogenous control. **(B)** Mature Oc. markers such as Acp5 and Ctsk are approx. 4-fold increased. The marker for Oc. progenitor cells Sfip1 (Pu.1) is also 4-fold elevated. Gapdh expression was used as endogenous control.
