# Supplementary material for: Loss of murine Gfi1 causes neutropenia and induces osteoporosis depending on the pathogen load and systemic inflammation
Source: PLoS One. 2018 Jun 7;13(6):e0198510. doi: 10.1371/journal.pone.0198510 (PMC5991660; doi:10.1371/journal.pone.0198510)
Supplement: S5 Fig — (DOCX) [file pone.0198510.s005.docx]

**S5 Figure**


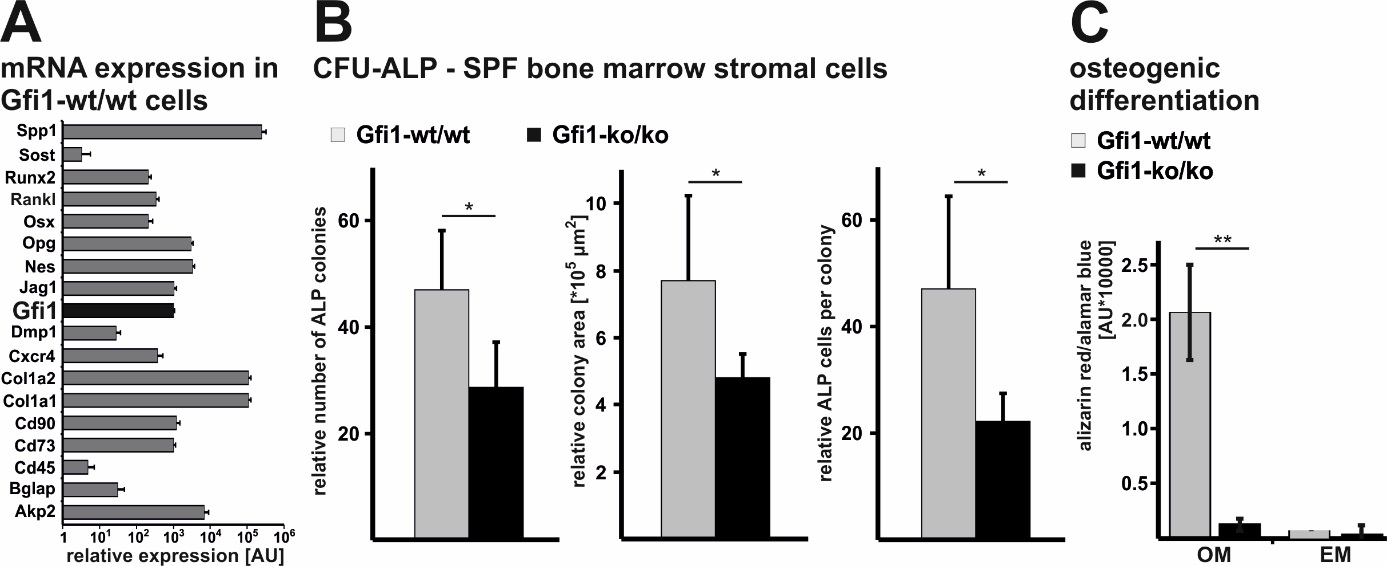


**S5 Figure. Gfi1 is expressed in mesenchymal stromal cells (MSC), promotes the MSC pool *in vitro* and is required for osteogenic *in vitro* differentiation.**

**(A)** Expression of Gfi1 mRNA was tested in lysates of *in vitro* cultured MSCs by qPCR. MSCs were cultured from wild-type control mice (n=5 with 3 technical replicates/sample). Gfi1 shows considerable expression in MSCs similar to levels of the MSC marker Cd73, Cd90, or Nestin. Specificity of *in vitro* cultured MSCs is indicated by very low levels of the B-lymphocyte marker Cd45 and the osteocyte specific mRNAs Sost as well as Dmp1. Gapdh expression was used as endogenous control. Please note logarithmic scaling of relative expression units. **(B)** Quantitative evaluation of alkaline phosphatase positive colony forming units (CFU-ALP) after bone marrow *in vitro* culture of control (n=5 with 3 technical replicates/sample) and Gfi1-ko/ko (n=4 with 3 technical replicates/sample) cells derived from animals housed at SPF. All evaluated parameters such as relative number of CFU-ALP colonies, colony area, and number of ALP positive cells per colony are significantly decreased in cultures of Gfi1-ko/ko cells. **(C)** Osteogenic differentiation of SPF MSCs Gfi1-wt/wt and Gfi1-ko/ko (each n=3 with 3 technical replicates/sample) occurred in osteogenic medium. Differentiated cells were stained with alizarin red to detect calcification and normalized to alamar blue staining. As negative control cells were cultured in expansion medium and analyzed. Error bars represent SD and statistical significance was calculated with t-test, * p ≤ 0.05 ** p ≤ 0.01.
