# Supplementary material for: Loss of murine Gfi1 causes neutropenia and induces osteoporosis depending on the pathogen load and systemic inflammation
Source: PLoS One. 2018 Jun 7;13(6):e0198510. doi: 10.1371/journal.pone.0198510 (PMC5991660; doi:10.1371/journal.pone.0198510)
Supplement: S6 Fig — (DOCX) [file pone.0198510.s006.docx]

**S6 Figure**


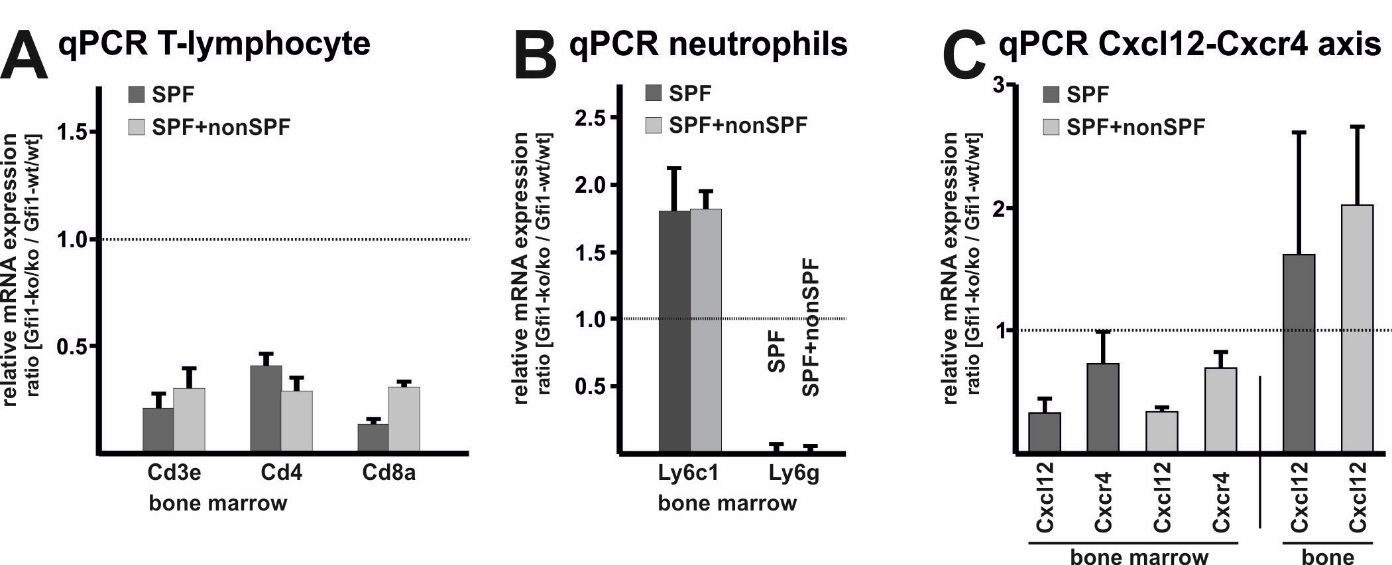


**S6 Figure. qPCR expression analysis of Cxcl12/Cxcr4 and immune cell marker genes.**

Expression of target mRNA from bone and bone marrow was measured with qPCR and results are shown as ratio Gfi1-ko/ko vs. Gfi1-wt/wt. Gapdh expression was used as endogenous control.  **(A)** All markers of lymphocyte production Cd3e, Cd4, and Cd8a demonstrate lower expression in Gfi1-ko/ko bone marrow. **(B)** The expression of Ly6c1 and Ly6g mRNA was analyzed to assess neutrophil differentiation. **(C)** Expression of Cxcl12 is approx. 2-fold elevated in cortical bone tissue upon SPF and SPF+nonSPF breeding. Conversely, in bone marrow Cxcl12 expression is downregulated. Cxcr4 levels appear mildly reduced.
