# Supplementary material for: Loss of murine Gfi1 causes neutropenia and induces osteoporosis depending on the pathogen load and systemic inflammation
Source: PLoS One. 2018 Jun 7;13(6):e0198510. doi: 10.1371/journal.pone.0198510 (PMC5991660; doi:10.1371/journal.pone.0198510)
Supplement: S1 Table — (DOCX) [file pone.0198510.s007.docx]

S1 Table: Health monitoring of mice kept under nonSPF, SPF, and SPF+nonSPF conditions.

| **Pathogen** | **Test frequency** | **nonSPF facility 1** | **SPF facility 2** | **SPF+nonSPF facility 3** |
| --- | --- | --- | --- | --- |
|  |  |  |  |  |
| **Viruses** |  |  |  |  |
| Mouse hepatitis virus (MHV) | 3 month | negative | negative | negative |
| Mouse rota virus (EDIM) | 3 month | negative | negative | negative |
| Minute virus of mice (MVM) | 3 month | negative | negative | negative |
| Mouse Parvoviruses (MPV) | 3 month | negative | negative | negative |
| Pneumonia virus of mice (PVM) | 3 month | negative | negative | negative |
| Sendai virus | 3 month | negative | negative | negative |
| Theiler´s murine encephalo-myelitis virus | 3 month | negative | negative | negative |
| Ectromelia virus (EV) | annually | negative | negative | negative |
| Lymphocyte choriomeningitis virus | annually | negative | negative | negative |
| Mouse adenovirus type 1 (FL) | annually | negative | negative | negative |
| Mouse adenovirus type 2 (K87) | annually | negative | negative | negative |
| Mouse cytomegalo type 1 (MCMV) | annually | negative | negative | negative |
| Reovirus type 3 | annually | negative | negative | negative |
| Mouse Norovirus |  | not tested | **occasionally positive** | not tested |
|  |  |  |  |  |
| **Bacteria, mycoplasma and fungi** | |  |  |  |
| Citrobacter rodentium | 3 month | negative | negative | negative |
| Clostridium piliforme | 3 month | negative | negative | negative |
| Corynebacterium kutscheri | 3 month | negative | negative | negative |
| Mycoplasma spp. | 3 month | negative | negative | negative |
| Pasteurellaceae | 3 month | **positive** | negative | **positive** |
| Pasteurella pneumotropica | 3 month | **positive** | negative | **positive** |
| Salmonella spp. | 3 month | negative | negative | negative |
| Streptococci β-haemolytic | 3 month | negative | negative | negative |
| Steptococcus pneumoniae | 3 month | negative | negative | negative |
| Heliobacter spp. | annually | **positive** | **occasionally positive** | **positive** |
| H. hepaticus | annually | not tested | negative | not tested |
| H. rodentium | annually | not tested | negative | not tested |
| H. billis | annually | not tested | negative | not tested |
| Pneumocystis carinii | annually | not tested | negative | not tested |
| Streptobacillus moniliformis | annually | negative | negative | negative |
|  |  |  |  |  |
| **Parasites** |  |  |  |  |
| Aspiculuris sp. | 3 month | negative | negative | negative |
| Syphacia sp. | 3 month | **occasionally positive** | negative | **occasionally positive** |
| Coccidia | 3 month | negative | negative | negative |
| Giardic | 3 month | negative | negative | negative |
| Spironucleus muris | 3 month | negative | negative | negative |
| Trichomonas sp. | 3 month | **positive** | not tested | **occasionally positive** |
| Flagella | 3 month | **positive** | not tested | negative |
| Arthropods | 3 month | negative | negative | negative |
| Endoparasites | 3 month | not tested | negative | **occasionally positive** |
| other protozoa | not tested | not tested | negative | not tested |

Explanation of values: negative - over time no positive animals were observed, positive - over time positive animals were frequently observed, occasionally positive - over time positive animals were sporadically observed.
