# Supplementary material for: Loss of murine Gfi1 causes neutropenia and induces osteoporosis depending on the pathogen load and systemic inflammation
Source: PLoS One. 2018 Jun 7;13(6):e0198510. doi: 10.1371/journal.pone.0198510 (PMC5991660; doi:10.1371/journal.pone.0198510)
Supplement: S2 Table — (DOCX) [file pone.0198510.s008.docx]

S2 Table: Volumetric microCT analysis of trabecular in vertebra of mice kept under nonSPF, SPF, and SPF+nonSPF conditions.

|  |  | **nonSPF conditions (6 weeks)** | | |  | **SPF conditions (8 weeks)** | | |  | **SPF+nonSPF conditions (9 weeks)** | | |
| --- | --- | --- | --- | --- | --- | --- | --- | --- | --- | --- | --- | --- |
| **value** | **unit** | **Gfi1-wt/wt** | **Gfi1-ko/ko** | **t-test** |  | **Gfi1-wt/wt** | **Gfi1-ko/ko** | **t-test** |  | **Gfi1-wt/wt** | **Gfi1-ko/ko** | **t-test** |
| **n** |  | 3 | 3 |  |  | 6 | 4 |  |  | 8 | 4 |  |
| **BV/TV** | % | 13.91 ± 1.63 | 5.57 ± 1.44 | p ≤ 0.01 |  | 7.53 ± 1.22 | 6.71 ± 1.42 | n.s. |  | 8.07 ± 1.02 | 5.16 ± 1.86 | p ≤ 0.01 |
| **BS/BV** | 1/mm | 61.46 ± 3.24 | 90.83 ± 5.15 | p ≤ 0.01 |  | 66.01 ± 5.52 | 78.66 ± 9.43 | p ≤ 0.05 |  | 62.38 ± 3.26 | 90.17 ± 18.83 | p ≤ 0.01 |
| **Tb.N** | 1/mm | 4.27 ± 0.54 | 2.55 ± 0.75 | p ≤ 0.05 |  | 1.49 ± 0.15 | 1.55 ± 0.19 | n.s. |  | 1.51 ± 0.15 | 1.26 ± 0.19 | p ≤ 0.05 |
| **Tb.Th** | mm | 0.033 ± 0.002 | 0.022 ± 0.001 | p ≤ 0.01 |  | 0.050 ± 0.004 | 0.043 ± 0.004 | p ≤ 0.05 |  | 0.053 ± 0.003 | 0.040 ± 0.009 | p ≤ 0.01 |
| **Tb.Sp** | mm | 0.204 ± 0.030 | 0.399 ± 0.148 | n.s. |  | 0.565 ± 0.054 | 0.630 ± 0.036 | n.s. |  | 0.615 ± 0.052 | 0.590 ± 0.050 | n.s. |
|  |  |  |  |  |  |  |  |  |  |  |  |  |

Statistical significance calculated by unpaired t-test of Gfi1-wt/wt vs. Gfi1-ko/ko for each breeding condition. All values are given from male mice as mean ± standard deviation. n.s. - not significant. Abbreviations: BV/TV - bone volume per tissue volume, BS/BV - bone surface per bone volume, Tb.N - trabecular number, Tb.Th - trabecular thickness, Tb.Sp - trabecular separation
