# Supplementary material for: Loss of murine Gfi1 causes neutropenia and induces osteoporosis depending on the pathogen load and systemic inflammation
Source: PLoS One. 2018 Jun 7;13(6):e0198510. doi: 10.1371/journal.pone.0198510 (PMC5991660; doi:10.1371/journal.pone.0198510)
Supplement: S3 Table — (DOCX) [file pone.0198510.s009.docx]

S3 Table: Histomorphometry in vertebra of Gfi1 mice kept under nonSPF, SPF, and SPF+nonSPF conditions.

|  |  | **nonSPF conditions (6 weeks)** | | |  | **SPF conditions (8 weeks)** | | |  | **SPF+nonSPF conditions (10 weeks)** | | |
| --- | --- | --- | --- | --- | --- | --- | --- | --- | --- | --- | --- | --- |
| **value** | **unit** | **Gfi1-wt/wt** | **Gfi1-ko/ko** | **t-test** |  | **Gfi1-wt/wt** | **Gfi1-ko/ko** | **t-test** |  | **Gfi1-wt/wt** | **Gfi1-ko/ko** | **t-test** |
| **n** |  | 5 | 4 |  |  | 5 | 4 |  |  | 4 | 4 |  |
| **BV/TV** | % | 19.39 ± 3.50 | 13.21 ± 2.27 | p ≤ 0.05 |  | 21.81 ± 2.57 | 21.09 ± 3.21 | n.s. |  | 21.95 ± 1.66 | 12.09 ± 3.86 | p ≤ 0.01 |
| **Tb.Th** | mm | 65.92 ± 6.87 | 50.98 ± 4.67 | p ≤ 0.01 |  | 72.33 ± 6.66 | 61.99 ± 4.57 | p ≤ 0.05 |  | 69.58 ± 3.28 | 51.05 ± 5.04 | p ≤ 0.01 |
| **Tb.N** | 1/mm | 2.93 ± 0.27 | 2.58 ± 0.23 | n.s. |  | 3.00 ± 0.10 | 3.39 ± 0.26 | n.s. |  | 3.16 ± 0.23 | 2.35 ± 0.59 | p ≤ 0.05 |
| **Tb.Sp** | µm | 278.08 ± 6.54 | 339.29 ± 38.24 | p ≤ 0.05 |  | 263.42 ± 5.33 | 234.43 ± 25.85 | n.s. |  | 248.48 ± 21.91 | 394.55 ± 109.03 | p ≤ 0.05 |
| **N.Ob/B.Pm** | 1/mm | 9.69 ± 2.79 | 4.43 ± 2.02 | p ≤ 0.05 |  | 11.97 ± 1.50 | 9.69 ± 2.01 | n.s. |  | 5.09 ± 1.36 | 8.30 ± 1.99 | p ≤ 0.05 |
| **Ob.S/BS** | % | 10.99 ± 3.50 | 4.77 ± 2.18 | p ≤ 0.05 |  | 13.71 ± 1.92 | 10.64 ± 2.33 | n.s. |  | 5.60 ± 1.48 | 9.37 ± 1.79 | p ≤ 0.05 |
| **N.Oc/B.Pm** | 1/mm | 0.92 ± 0.20 | 0.49 ± 0.05 | p ≤ 0.01 |  | 0.77 ± 0.28 | 1.29 ± 0.32 | p ≤ 0.05 |  | 0.58 ± 0.13 | 1.34 ± 0.40 | p ≤ 0.05 |
| **Oc.S/BS** | % | 1.19 ± 0.24 | 0.58 ± 0.08 | p ≤ 0.01 |  | 1.03 ± 0.35 | 1.97 ± 0.66 | p ≤ 0.05 |  | 0.74 ± 0.15 | 2.08 ± 0.42 | p ≤ 0.01 |
| **OV/BS** | % | 0.158 ± 0.062 | 0.163 ± 0.050 | n.s. |  | 0.323 ± 0.125 | 0.025 ± 0.010 | p ≤ 0.01 |  | 0.323 ± 0.245 | 0.233 ± 0.096 | n.s. |
| **OS/BS** | % | 3.763 ± 0.978 | 2.550 ± 0.652 | n.s. |  | 5.148 ± 2.459 | 1.085 ± 0.613 | p ≤ 0.05 |  | 3.748 ± 1.863 | 3.215 ± 0.586 | n.s. |
| **O.Th** | mm | 0.628 ± 0.075 | 0.843 ± 0.196 | n.s. |  | 1.055 ± 0.486 | 0.320 ± 0.165 | p ≤ 0.05 |  | 1.120 ± 0.150 | 0.830 ± 0.207 | n.s. |
|  |  |  |  |  |  |  |  |  |  |  |  |  |

Statistical significance calculated by unpaired t-test of Gfi1-wt/wt vs. Gfi1-ko/ko for each breeding condition. All values are given from male mice as mean ± standard deviation. n.s. - not significant. Abbreviations: BV/TV - bone volume/tissue volume, Tb.Th - trabecular thickness, Tb.N - trabecular number, Tb.Sp - trabecular separation, N.Ob/B.Pm - number of osteoblasts per bone perimeter, Ob.S/BS - osteoblast surface per bone surface, N.Oc/B.Pm - number of osteoclasts per bone perimeter, Oc.S/BS - osteoclast surface per bone surface, OV/BS - osteoid volume per bone bone surface, OS/BS - osteoid surface per bone surface, O.Th - osteoid thickness
