# Supplementary material for: Loss of murine Gfi1 causes neutropenia and induces osteoporosis depending on the pathogen load and systemic inflammation
Source: PLoS One. 2018 Jun 7;13(6):e0198510. doi: 10.1371/journal.pone.0198510 (PMC5991660; doi:10.1371/journal.pone.0198510)
Supplement: S4 Table — (DOCX) [file pone.0198510.s010.docx]

S4 Table: Differential peripheral blood cell count of mice kept under SPF and SPF+nonSPF conditions.

|  |  | **SPF condition** | |  |  | **SPF+nonSPF conditions** | | |  |
| --- | --- | --- | --- | --- | --- | --- | --- | --- | --- |
| **value** | **unit** | **Gfi1-wt/wt** | **Gfi1-ko/ko** | **t-test** |  | **Gfi1-wt/wt** | **Gfi1-ko/ko** | **t-test** |  |
| **n** |  | 13 | 14 |  |  | 11 | 11 |  |  |
| **neutrophils**  **(segmented)** | % | 14.77 ± 5.43 | 5.57 ± 3.06 | p ≤ 0.01 |  | 12.73 ± 6.02 | 4.09 ± 1.51 | p ≤ 0.01 |  |
| **neutrophils**  **(banded)** | % | 0.00 | 0.00 |  |  | 0.00 | 0.00 |  |  |
| **lymphocytes** | % | 84.31 ± 4.82 | 86.29 ±11.32 | n.s. |  | 86.18 ± 6.49 | 80.09 ± 22.51 | n.s. |  |
| **monocytes** | % | 0.15 ± 0.38 | 2.43 ± 2.53 | p ≤ 0.01 |  | 0.90 ± 0.99 | 2.27 ± 2.65 | n.s. |  |
| **eosinophiles** | % | 0.15 ± 0.38 | 0.36 ± 0.74 | n.s. |  | 0.00 | 0.36 ± 0.67 | n.s. |  |
| **basophiles** | % | 0.00 | 0.00 |  |  | 0.00 | 0.00 |  |  |
| **pathological forms** | % | 0.62 ± 2.22 | 5.14 ± 12.74 | n.s. |  | 0.00 | 13.18 ± 23.55 | p ≤ 0.01 |  |
|  |  |  |  |  |  |  |  |  | |

Statistical significance calculated by unpaired t-test of Gfi1-wt/wt vs. Gfi1-ko/ko mice. All values are given as mean ± standard deviation.

n.s. - not significant.
