# Supplementary material for: Loss of murine Gfi1 causes neutropenia and induces osteoporosis depending on the pathogen load and systemic inflammation
Source: PLoS One. 2018 Jun 7;13(6):e0198510. doi: 10.1371/journal.pone.0198510 (PMC5991660; doi:10.1371/journal.pone.0198510)
Supplement: S5 Table — (DOCX) [file pone.0198510.s011.docx]

S5 Table: Quantitative multiplex analysis for immunmodulatory cytokines of mice kept under nonSPF, SPF, and SPF+nonSPF conditions.

|  |  | **nonSPF** |  |  |  | **SPF** |  |  |  | **SPF+nonSPF** | |  |
| --- | --- | --- | --- | --- | --- | --- | --- | --- | --- | --- | --- | --- |
| **cytokine** | **unit** | **Gfi1-wt/wt** | **Gfi1-ko/ko** | **t-test p-value** |  | **Gfi1-wt/wt** | **Gfi1-ko/ko** | **t-test p-value** |  | **Gfi1-wt/wt** | **Gfi1-ko/ko** | **t-test p-value** |
| **n** |  | 3 pools | 2 pools |  |  | 7 | 5 |  |  | 10 | 5 |  |
| **Il-1a *** | pg/ml | 5.6 ± 2.9 | 247.5 ± 139.8 | n.a. |  | 6.1 ± 1.8 | 34.3 ± 21.8 | n.s |  | 10.7 ± 3.8 | 129.4 ± 42.7 | p ≤ 0.001 |
| **Il-1b *** | pg/ml | 173.0 ± 98.9 | 6426.2 ± 3785.2 | n.a. |  | 44.3 ± 28.5 | 330.3 ± 126.5 | p ≤ 0.05 |  | 251.4 ± 77.3 | 3379.2 ± 974.9 | p ≤ 0.001 |
| **Il-2** | pg/ml | 4.05 ± 4.1 | 230.7 ± 85.4 | n.a. |  | b.l.d. | 48.1 ± 21.8 | p ≤ 0.05 |  | b.l.d. | 59.0 ± 18.9 | p ≤ 0.001 |
| **Il-3** | pg/ml | 3.7 ± 1.3 | 55.5 ± 52.3 | n.a. |  | 2.1 ± 0.3 | 11.4 ± 4.5 | p ≤ 0.05 |  | 2.4 ± 0.8 | 47.8 ± 22.7 | p ≤ 0.05 |
| **Il-5** | pg/ml | 37.6 ± 19.1 | 329.1 ± 256.1 | n.a. |  | 12.5 ± 8.2 | 62.0 ± 40.1 | n.s. |  | 12.2 ± 11.1 | 200.5 ± 98.6 | p ≤ 0.05 |
| **Il-6 *** | pg/ml | 22.2 ± 11.3 | 903.9 ± 803.0 | n.a. |  | 11.1 ± 3.0 | 144.9 ± 106.0 | n.s. |  | 14.7 ± 9.6 | 372.8 ± 203.5 | p ≤ 0.05 |
| **Il-10** | pg/ml | b.l.d. | b.l.d. | n.a. |  | b.l.d. | b.l.d. | n.a. |  | b.l.d. | b.l.d. | n.a. |
| **Il-12** | pg/ml | 190.3 ± 77.8 | 4082.9 ± 3345.6 | n.a. |  | 47.3 ± 13.3 | 379.7 ± 222.0 | n.s. |  | 196.9 ± 143.4 | 3926.9 ± 2265.4 | p ≤ 0.05 |
| **Il-17** | pg/ml | 37.1 ± 37.1 | 469.7 ± 309.2 | n.a. |  | b.l.d. | 69.1 ± 35.3 | p ≤ 0.05 |  | b.l.d. | 106.8 ± 36.6 | p ≤ 0.001 |
| **MCP-1** | pg/ml | 67.2 ± 67.2 | 2078.6 ± 968.7 | n.a. |  | 54.3 ± 36.2 | 334.4 ± 86.3 | p ≤ 0.01 |  | 194.6 ± 46.0 | 1278.2 ± 537.9 | p ≤ 0.05 |
| **IFNγ** | pg/ml | b.l.d. | 26.8 ± 26.8 | n.a. |  | b.l.d. | 6.8 ± 3.6 | p ≤ 0.05 |  | b.l.d. | 32.9 ± 18.4 | p ≤ 0.05 |
| **TNFα *** | pg/ml | 20.6 ± 10.4 | 261.8 ± 38.5 | n.a. |  | 8.9 ± 3.4 | 44.6 ± 18.8 | n.s. |  | 29.3 ± 7.1 | 198.4 ± 48.5 | p ≤ 0.001 |
| **MIP-1a** | pg/ml | b.l.d. | b.l.d. | n.a. |  | b.l.d. | b.l.d. | n.a. |  | b.l.d. | b.l.d. | n.a. |
| **RANTES** | pg/ml | 26.9 ± 8.6 | 18.3 ± 6.1 | n.a. |  | 47.4 ± 5.6 | 29.1 ± 5.8 | n.s. |  | 33.4 ± 4.9 | 25.8 ± 5.3 | n.s. |
|  |  |  |  |  |  |  |  |  |  |  |  |  |

Statistical significance was calculated by unpaired t-test of Gfi1-wt/wt vs. Gfi1-ko/ko mice. All values are given as mean ± standard error of the mean. For nonSPF conditions we compared 3 pools of Gfi1-wt/wt and 2 pools of Gfi1-ko/ko mice (n=4 animals/pool). Due to the low number of samples we did not calculate the statistics. * - cytokines most relevant for bone, n.s. - not significant, b.l.d. - below limit of detection, n.a. - not statistical analyzed.
